# Smaller hamstrings autograft size after primary ACL reconstruction is associated with higher odds for graft failure: A meta‐analysis on autografts sizes covering 46,268 patients

**DOI:** 10.1002/ksa.70069

**Published:** 2025-09-22

**Authors:** Rebecca Hamrin Senorski, Johan Högberg, Kevin Teow, Anna Nordenholm, Janina Kaarre, Thorkell Snaebjörnsson, Volker Musahl, Kristian Samuelsson, Eric Hamrin Senorski

**Affiliations:** ^1^ Department of Health and Rehabilitation, Unit of Physiotherapy, Institute of Neuroscience and Physiology, Sahlgrenska Academy University of Gothenburg Gothenburg Sweden; ^2^ Sahlgrenska Sports Medicine Center Gothenburg Sweden; ^3^ Department of Orthopaedics, Institute of Clinical Sciences, Sahlgrenska Academy University of Gothenburg Gothenburg Sweden; ^4^ Department of Orthopaedic Surgery, UPMC Freddie Fu Sports Medicine Center University of Pittsburgh Medical Center Pittsburgh Pennsylvania USA; ^5^ Department of Orthopaedics, Landspitali, University Hospital of Iceland, Reykjavik, Iceland; Medical Faculty University of Iceland Reykjavik Iceland; ^6^ Department of Orthopaedics Sahlgrenska University Hospital Mölndal Sweden

**Keywords:** autograft, graft diameter, hamstrings tendon, patellar tendon, revision, re‐rupture

## Abstract

**Purpose:**

To investigate the association between autograft size and graft failure for hamstrings tendon (HT), patellar tendon (PT) and quadriceps tendon (QT) autografts.

**Methods:**

Medline, PubMed, Cochrane Library, Embase, Amed and Web of Science were searched at four separate time points, with the most recent search in February 2025. Eligible studies included patients with primary anterior cruciate ligament reconstruction (ACLR) with (graft failures) or without (survivals) graft failure who had specified autograft size for primary ACLR. Graft failure was defined as a re‐rupture of the reconstructed ACL and/or positive pivot shift. Standardised mean differences were calculated for continuous variables, and odds ratios expressed with 95% confidence interval for dichotomous variables of autograft size for survivals versus graft failures. Risk of bias was assessed with RoBANS 2/RoB2 and certainty of evidence with GRADE.

**Results:**

In total, 46,268 patients, of which 43,660 HT autograft, 2410 PT autograft and 198 QT autograft were covered in 35 studies. An HT autograft size of < 7 mm had 81% greater odds for a graft failure compared to ≥ 7 mm (*p* = 0.01), <8 mm HT autograft size had 46% greater odds for a graft failure compared to ≥ 8 mm (*p* < 0.0001), <9 mm HT autograft size had 34% greater odds for a graft failure compared to ≥ 9 mm (*p* = 0.001). No significant odds for a graft failure were observed for patients with ≥ 10 mm HT autograft size compared to < 10 mm, or for patients with PT autograft. Only two of the included studies provided data on the QT autograft where none presented standard deviation, thus these studies could not be pooled and were presented qualitatively.

**Conclusion:**

Patients treated with a smaller HT autograft size have greater odds for a graft failure compared to patients with a greater autograft size. There was no association between autograft size for patients treated with PT autograft and graft failure. Surgeons should consider autograft size when performing ACLR as the size of HT autografts influences the risk of a graft failure.

**Level of Evidence:**

Level IV.

**Abbreviations**
ACL: anterior cruciate ligament
ACLR: anterior cruciate ligament reconstruction
AMED: Allied and Complementary Medicine Database
CI: confidence interval
Embase: Excerpta Medica Database
GRADE: Grading of Recommendations Assessment Development and Evaluation methodology
HT: hamstrings tendon
LOE: level of evidence
Medline: Medical Literature Analysis and Retrieval System Online
MeSH: Medical Subject Headings
Mm: millimetres
MRI: magnetic resonance imaging
n: number
OR: odds ratio
PRISMA: Preferred Reporting Items for Systematic Reviews and Meta‐Analyses
PT: patellar tendon
QT: quadriceps tendon
RCT: randomised controlled trial
RoB: risk of bias assessment tool for randomised controlled trials
RoBANS: risk of bias assessment tool for non‐randomised controlled trials
SD: standard deviation
SMD: standardised mean difference

## INTRODUCTION

An anterior cruciate ligament (ACL) injury is a significant knee trauma often experienced by athletes who participate in sports [62]. The injury situation is typically non‐contact in low degrees of knee flexion (20°–40°) with dynamic knee valgus load [20]. Treatment consists of rehabilitation with or without surgical ACL reconstruction (ACLR) [21]. Graft selection for ACLR should be individualised depending on patient history (e.g., number of ruptures), characteristics (e.g., knee laxity), type of sports played (e.g., hamstring or quadriceps dominant) and surgeons experience [58]. To date, the hamstring tendon (HT) autograft is most commonly used, representing about 50% of all primary ACLR globally, followed by the patellar tendon (PT) autograft 40% and quadriceps tendon (QT) autograft 10% [4]. Studies, however, indicate that HT autografts exhibit greater residual anterior tibial translation [36], slower graft healing [75], and higher graft failure rates compared to PT autograft [12, 61, 80]. On the other hand, the QT autograft has been reported with superior functional outcomes compared with the HT, and less harvest site pain and similar survival rates as the PT autograft [51]. One systematic review suggested that results from previous randomised controlled trials (RCT) comparing the HT‐ and PT autografts are statistically fragile, as small changes in outcomes or accounting for patients lost to follow‐up could reverse their conclusions [42]. Another study that compared outcomes between the QT, HT and PT autografts reported low certainty of evidence for functional performance and patient reported outcomes which limits the confidence in these findings [19].

To ensure acceptable strength and stiffness of the autograft, it is important to select a sufficient graft diameter/width with a graft sizing block. Graft diameter/width (collectively autograft size) has been suggested as a predictor for graft failure, defined as a re‐rupture of the reconstructed ACL and/or positive pivot shift, where HT autograft diameter < 8 mm has been reported to increase risk for graft failure compared to ≥ 8 mm [2, 67, 70]. Although there are conflicting evidence on the effect of autograft sizes impact on graft failure [29, 69]. Prior to this study, four systematic reviews have been conducted on autograft size and the risk for second ACL injury [3, 14, 30, 34]. However, existing reviews have primarily focused on HT autografts and specific age groups, highlighting the need for a comprehensive systematic review that includes all age populations and compares autograft size for both HT and PT autografts to better guide individualised graft selection in ACL reconstruction. The objective of this systematic review and meta‐analysis was to investigate the association between autograft size and graft failure for HT, PT and QT autografts. Our hypothesis was that patients treated with a larger autograft size would have lower odds for graft failure compared to smaller autograft size.

## METHODS

This systematic review and meta‐analysis was reported following the Preferred Reporting Items for Systematic Reviews and Meta‐Analyses (PRISMA) guidelines [72]. This systematic review was prospectively registered on PROSPERO with registration ID: CRD42022307163.

### Eligibility criteria

Eligible studies included randomised controlled trials (RCTs), longitudinal cohort studies, and case–controls. Studies written in English, Swedish, Finnish, Norwegian or Danish, including patients who had undergone primary ACLR with either HT, PT or QT autografts, with or without subsequent graft failure, and had specified autograft size for primary ACLR were included. Studies published as opinion pieces, technical notes, editorials, reviews or cadaver and animal studies were excluded. The first search was performed in February 2022, updated in November 2022, November 2023 and February 2025.

### Information sources and search strategies

A comprehensive systematic search was performed by a medical librarian at the Biomedical library at the University of Gothenburg using Medical Literature Analysis and Retrieval System Online (Medline), PubMed, Cochrane Library, Excerpta Medica database (EMBASE), Allied and Complementary Medicine Database (AMED) and Web of Science. The search combined the use of Medical Subject Headings (MeSH) with free text terms (Supporting Information: File S1).

### Study selection process

Studies were systematically assessed for eligibility for inclusion by screening titles and abstracts using the Rayyan QCRI web application for systematic reviews [54]. Four authors (R.S., J.H., A.N. and K.T.) performed the screening of titles and abstracts for eligibility independently. Authors were blinded to each other until initial screening based on titles and abstracts was done. Cohen's kappa coefficient of agreement was calculated [41] after the initial inclusion/exclusion process was finalised, which demonstrated a moderate to perfect agreement (Cohen's kappa coefficient = 0.51–0.90). Eligible studies assessed by screening titles and abstracts were then read in full text before the decision of inclusion. A consensus discussion was held between the screening authors in collaboration with the senior author (E.H.S.) in the event of disagreement.

Studies with missing data, such as autograft size for patients without graft failure (survivals), were contacted by e‐mail for additional data. Forty‐two corresponding authors were contacted, three of these were excluded after email response due to not having the requested data, 25 were excluded due to no response, and 14 studies were included after providing the missing additional data [16, 18, 28, 44, 48, 49, 50, 60, 63, 67, 68, 76, 77, 78], however, two were excluded due to using the same dataset, such as the Swedish National Knee Ligament Registry [16, 68], thus, the study with the largest cohort was used [67]. Furthermore, one study was later excluded for the HT analysis due to using a matched cohort [76].

Five studies [1, 28, 49, 60, 63] presented demographical data for the entire cohort together, however, separate data for graft failures and survivals was provided and could therefore be included. Due to limited data on QT autograft [28, 60], pooling was not possible. Hence data on QT autograft was presented qualitatively in a free text.

### Data collection

Two authors (R.S. and K.T.) extracted data from the included studies into Excel (version 16; Microsoft Corporation, Redmond, Washington, USA). Extracted data consisted of author, title, journal, year of publication, study design, purpose, sample size, groups (survivals and graft failures), sex, age, autograft size (mm) for survivals/failures, graft type (e.g., HT autograft), loss to follow‐up, the definition of graft failure, number of graft failures, time (months) of graft failure from ACLR, follow up time (months) from ACLR, method of analysis, operative procedure (e.g., anterofemoral or transtibial portal), confounders that each study has expressed and adjusted for in their analysis and conclusion.

### Data items

Primary outcome of interest in this systematic review and meta‐analysis was the association between autograft size and graft failure. Autograft size and number of patients were recognised for (1) survivals and (2) graft failures. Graft failure was defined as ACL revision, graft rupture, re‐rupture, graft failure assessed by magnetic resonance imaging (MRI), or/and a positive pivot shift in the included studies, depending on the definition for each study.

### Risk of bias assessment

The risk of bias was assessed using the revised Risk of Bias Assessment tool for Non randomised Studies (RoBANS 2) [37]. Two authors (RHS and AN) assessed the risk of bias for included studies independently. Any disagreement was solved by consensus discussions between the assessment authors and the senior author (E.H.S.).

The RoBANS 2 consists of eight domains: (a) comparability of target group, (b) target group selection, (c) confounders, (d) measurement of intervention/exposure, (e) blinding of assessors (f) outcome assessment, (g) incomplete outcome data and (h) selective outcome reporting [64]. A respective domain is assessed separately, and rated as low, high or unclear risk of bias. RoBANS 2 has fair to moderate inter‐rater reliability (weighted kappa 0.25–0.49).

For the included RCTs, the revised Cochrane Risk of Bias tool (RoB 2) tool was used. RoB 2 consists of five domains which are, bias arising from the randomisation process, bias due to deviations from intended interventions, bias due to missing outcome data, bias in measurement of the outcome, bias in selection of the reported result, and overall bias. Each domain is graded with yes, probably yes, probably no, no, and no information. The risk of bias is judged based on the overall risk for each domain. The risk of bias for all domains is then summarised for each study. The RoB 2 is interpreted as, low risk of bias if all domains are considered low risk of bias, some concerns if no domain was judged as high risk, and at least one domain as some concerns, high risk if at least one domain was judged as high risk of bias or if several domains were regarded as some concerns [71].

### Effect measures

Standardised mean differences were calculated for continuous variables and odds ratios for dichotomous analysis of autograft size for survivors versus graft failures. Standardised mean difference was expressed as Hedges' g, with the following reference values: 0.20 = small, 0.50 = medium and 0.80 = large [13]. For the dichotomous analysis, autograft size was expressed as ≥ versus < or, > versus ≤ a specific autograft size. The standardised mean differences and odds ratios (ORs) were expressed with a 95% confidence interval (CI).

### Synthesis methods

Data was structured by (1) mean autograft size for survivals and for graft failures, and (2) autograft size as dichotomous, that is, ≥/< or ≤/> for survivals or graft failures. Sub‐analyses were made based on age, where studies with a cohort of patients with mean age ≤18 years old were analysed separately. Analyses were structured by graft type, that is, HT and PT autografts. Standardised mean differences and ORs were calculated and pooled using a random effects model with the inverse variance method. Results were expressed with 95% CI and visualised in forest plots using the Review Manager 5.4 (RevMan, version 5.4.1, Cochrane, London, UK) by authors RHS and JH. Due to heterogeneity in the definition of graft failures, for example, defined by MRI or by a positive pivot shift, an analysis with standardised mean difference was chosen to perform the continuous analysis as effect measure despite all included studies reported autograft size in mm. A random effects model with inverse variance was chosen due to the clinical heterogeneity in the included studies and determined by discussion between authors.

Statistical heterogeneity was assessed with the *I* ^2^ index for each analysis categorised as no heterogeneity 0.0%–24.9%, low 25.0%–49.9%, moderate 50.0%–74.9% and high 75.0%–100.0% [27].

If the standard deviation (SD) for autograft size was not presented in the included study, an estimated SD was calculated based on the SD of the other included studies [22]. Due to that four studies in the HT autograft analyses [23, 33, 35, 49] and three studies in the PT autograft analyses [23, 28, 65] did not presented SD, a sensitivity analysis was made excluding these studies without SD to determine their impact on results (Supporting Information: File S2).

### Certainty of evidence

Certainty of evidence for the outcomes of interest in this systematic review was assessed by the first (RHS) and fifth (AN) authors, using the Grading of Recommendations Assessment Development and Evaluation methodology (GRADE) [6]. The GRADE firstly considers study design, where randomised controlled trials (RCTs) are considered as starting assessment as high, with grade 4, and observational studies are considered starting assessment as low, with Grade 2. Furthermore, the GRADE values the risk of bias, inconsistency, indirectness, imprecision, and risk for publication bias. Each domain included in the GRADE may result in downgrading the starting grade (Grade 4 or 2) by one or two points depending on the degree of study limitation for the respective domain.

- Study design constitutes the starting point and can start on three or four for RCTs or one or two for cohort studies.
- Risk of bias, a downgrade can be made if >75% of included studies have a high or very high risk of bias.
- Inconsistency, a downgrade can be made if analyses have a large heterogeneity based on the *I* ^2^ factor for each analysis.
- Indirectness, a downgrade can be made if the included population inadequately reflects the target population of the present review.
- Imprecision, a downgrade can be made if the confidence interval is deemed too wide which can imply that results are not as trustworthy.
- Publication bias downgrade can be made if there is an asymmetry visualised in the funnel plot.

GRADE also has the possibility of upgrading analyses by three separate questions for each analysis, that is, large effect (+1 large, +2 very large), dose‐response (+1 evidence of a gradient), and all plausible residual confounding (+1 would reduce a demonstrated effect, +1 would suggest a spurious effect if no effect was observed). The judgement of the upgrade was made after the initial domains were determined. Collectively, based on the assessment described above, the certainty of evidence for respective outcomes results in a very low, low, moderate, or high certainty of evidence.

## RESULTS

### Study selection

**Figure 1:**
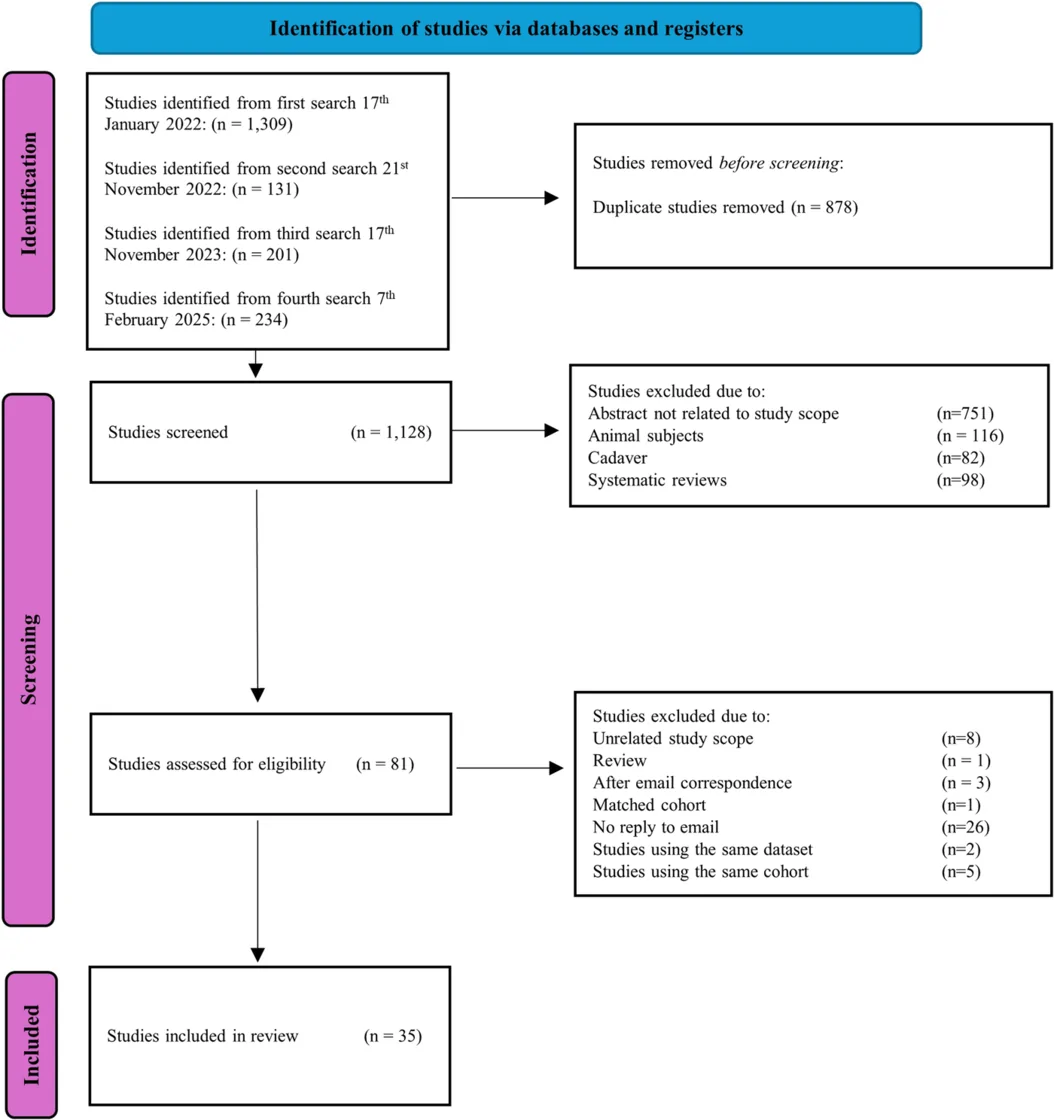
The Preferred Reporting Items for Systematic Reviews and Meta‐Analysis (PRISMA) flow chart of the selection process.

The literature search was performed in six databases on four separate occasions, yielding 2006 studies. After removing duplicates, 1128 studies were screened by abstract and title, with 81 studies selected for full‐text review. Number of included studies per electronic platform for all four searches is presented in Supporting Information: Appendix Figure 1. Finally, 35 studies were included. Figure 1 displays a detailed description of the selection process.

### Characteristics of included studies

Included studies were published between 1998 and 2025. Twenty‐six studies presented data for HT autografts, five studies for both HT and PT autografts, one for HT and QT, one for PT and QT autografts, two studies for PT autograft. In total, included studies covered 46,268 patients, of which 43,660 were treated with HT autograft, 2410 treated with PT autograft and, 198 treated with QT autograft. Age of patients in included studies ranged between 11 and 62 years, whereas five included studies only had data on adolescents with a mean age ≤ 18 years old [25, 46, 48, 50, 52]. Graft failures ranged between 2% at the lowest [24, 67, 77], to 50% at the highest [76], with follow‐ups between 6 months [43] and 147 months [50] after ACLR. Table 1 presents the characteristics of included studies.

**Table 1:** Characteristics of included studies.

Study	LOE	Age (years)	*n* 2nd ACL	*n* Survivals	Autograft size, survivals	Autograft size, 2nd ACL	Follow‐up (months) after ACLR
Hamstrings tendon autograft							
Gudas et al. [23]	III	25.1	14	81	8 mm*	8 mm*	≥24
Jagadeesh et al. [31]	IV	30.89 ± 9.3	9	135	8.40 ± 0.56* ≤7 mm = 10 7.01–8.0 mm = 88 8.1–9.0 mm = 37 >9 mm = 11	7.78 ± 0.67* ≤7 mm = 3 7.01–8.0 mm = 5 8.1–9.0 mm = 1 >9 mm = 0	Mean 18.4 ± 4.5
Gupta et al. [24]	III	25.14 ± 5.74	2	101	≥8 mm** = 34 <8 mm = 67	≥8 mm** = 0 <8 mm = 2	Mean 22 (range 12–34)
Murgier et al. [52]	III	17.5	44	637	6 mm = 5*** 6.5 mm = 8 7.0 mm = 64 7.5 mm = 107 8.0 mm = 144 8.5 mm = 133 9.0 mm = 101 9.5 mm = 45 10 mm = 27 10.5 mm = 2 11 mm = 3	6 mm = 0*** 6.5 mm = 0 7.0 mm = 6 7.5 mm = 6 8.0 mm = 9 8.5 mm = 8 9.0 mm = 12 9.5 mm = 1 10 mm = 0 10.5 mm = 0 11 mm = 0	Mean 38.6 ± 10.7
Wernecke et al. [79]	IV	<9 mm = 27.3 ± 9.7 9 mm = 21.5 ± 10 8.5 mm = 26.9 ± 9.8 8 mm = 30.0 ± 9.7 7.5 mm = 30.4 ± 10.7 7 mm = 31.6 ± 10.5 6.5 mm = 28.2 ± 10.2 <6.5 mm = 25.9 ± 10.4	44	739	>9 mm = 28*** 9 mm = 63 8.5 mm = 109 8 mm = 226 7.5 mm = 158 7 mm = 116 6.5 mm = 27 <6.5 mm = 12	>9 mm = 3*** 9 mm = 4 8.5 mm = 3 8 mm = 13 7.5 mm = 10 7 mm = 6 6.5 mm = 4 <6.5 mm = 1	Mean 39.48 ± 14.4
Keuning et al. [35]	III	2ACL 24.3 S 29.4	41	606	8.1 mm*	8.2 mm*	Mean 66 ± 18
Rahardja et al. [57]	II	2ACL 24.1 ± 8.5 S 30.4 ± 11.1	242	5574	8.4 ± 0.8,* <8 mm = 1011** ≥8 mm = 4563	8.3 ± 0.8* <8 mm = 59** ≥8 mm = 183	Mean 40.8 ± 16.8
Mariscalco et al. [47]	III	≤8 mm = 21 (17–28.5) >8 mm = 27 (19.8–35.2)	14	249	>8 mm** = 64 ≤8 mm = 185	<8 mm** = 0 ≤8 mm = 14	24
Marigi et al. [46]	IV	16 (15–18)	9	34	≤7 mm = 3 > 7 mm = 31 <8 mm = 6 ≥ 8 mm = 28 <9 mm = 18 ≥ 9 mm = 16	≤7 mm = 3,*** >7 mm = 6 <8 mm = 4 ≥8 mm = 5 <9 mm = 7 ≥9 mm = 2	Median 70.8 (range 46.8–123.6)
Snaebjornsson et al. [67]	III	At injury 24.9 (9.3) At reconstruction 26.8 (9.7)	356	16,740	<7 mm = 257*** ≥7 mm = 16,483 <8 mm = 4121 ≥8 mm = 12,619 <9 mm = 12,400 ≥9 mm = 4340 <10 mm = 16,092 ≥10 mm = 648	<7 mm = 7*** ≥7 mm = 349 <8 mm = 115 ≥8 mm = 241 <9 mm = 287 ≥9 mm = 69 <10 mm = 349 ≥10 mm = 7	24
Magnussen et al. [43]	III	25.0 ± 10.5 (11–52)	18	238	<7 mm = 4*** 7 mm = 47 7.5 mm = 30 8 mm = 100 8.5 mm = 13 9 mm = 38 >9 mm = 6	<7 mm = 2*** 7 mm = 6 7.5 mm = 2 8 mm = 7 8.5 mm = 0 9 mm = 1 >9 mm = 0	Mean 14 ± 8 (range 6–47)
Tang et al. [73]	III	27.3 ± 8.1 (13–55)	20	374	<7 mm = 16* 7 mm = 83 7.5 mm = 54 8 mm = 159 8.5 mm = 32 9 mm = 25 >9 mm = 5	<7 mm = 1*** 7 mm = 6 7.5 mm = 9 8 mm = 4 8.5 mm = 0 9 mm = 0 >9 mm = 0	n.r
Spragg et al. [69]	III	2ACL 17.6 (15.9‐20.4) Control 17.6 (15.9–20.4)	124	367	8.1 ± 0.73* 6 mm = 1*** 6.5 mm = 4 7.0 mm = 55 7.5 mm = 38 8 mm = 165 8.5 mm = 22 9.0 mm = 70 9.5 mm = 2 10 mm = 10	7.9 ± 0.75* 6 mm = 2*** 6.5 mm = 2 7.0 mm = 27 7.5 mm = 15 8 mm = 53 8.5 mm = 7 9.0 mm = 15 9.5 mm = 0 10 mm = 3	Median 22.8 (IQrange 14.4–39.6)
Kamien et al. [33]	III	≤25 years >25 years	15	80	8.54 mm* 6.5 mm = 0 7 mm = 8 7.5 mm = 3 8 mm = 24 8.5 mm = 5 9 mm = 29 9.5 mm = 4 10 mm = 5 10.5 mm = 1 11 mm = 1	8.43 mm* 6.5 mm = 1 7 mm = 2 7.5 mm = 0 8 mm = 5 8.5 mm = 0 9 mm = 4 9.5 mm = 0 10 mm = 3 10.5 mm = 0 11 mm = 0	≥24
Park et al. [55]	IV	29.8 ± 10.7 (14–61)	12	298	4.5 mm = 1*** 5.5 mm = 4 6.0 mm = 24 6.5 mm = 48 7.0 mm = 66 7.5 mm = 83 8.0 mm = 49 8.5 mm = 19 9.0 mm = 3 10.0 mm = 1	6 mm = 2*** 6.5 mm = 2 7.0 mm = 5 7.5 mm = 3 ≥7.5 mm = 3 <8 mm = 12 ≥8 mm = 0	Mean 53.9 ± 8.8 (range 24.0–70.8)
Inderhaug et al. [29]	II	29.1 ± 10.7	150	3,879	≤7 mm = 332*** 7.5 mm = 480 8.0 mm = 1652 8.5 mm = 464 9.0 mm = 756 9.5 mm = 55 ≥10.0 mm = 140	≤7 mm = 11*** 7.5 mm = 21 8.0 mm = 74 8.5 mm = 11 9.0 mm = 29 9.5 mm = 0 ≥10.0 mm = 4	Mean 30 ± 15.6
Schlumberger et al. [63]	IV	32.4 ± 12.2	73	2375	7.9 ± 0.6 mm	7.8 ± 0.6 mm	Mean 60 ± 13.2 (range 36–84)
Jeffers et al. [32]	IV	n.r	2	31	8 mm = 6 8.5 mm = 10 9 mm = 11 9.5 mm = 4	8 mm = 1 8.5 mm = 0 9.0 mm = 0 9.5 mm = 1*	n.r
Yamanashi et al. [81]	III	S 27.0 ± 12.2 2ACL 18.6 ± 6.0	27	205	8.8 ± 0.7*	8.5 ± 0.7*	Mean 18.9 ± 9.7
Wan et al. [77]	II	5 strand: 29. ± 8.7 4 strand: 27.7 ± 8.5	1	44	6 mm = 1 6.5 mm = 0 7 mm = 9 7.5 mm = 2 8 mm = 10 8.5 mm = 4 9 mm = 12 9.5 mm = 3 10 mm = 3	7 mm = 1	Mean 5 strand 17.5 ± 2.34 Mean 4 strand 17.4 ± 2.57
Manara et al. [44]	III	30 (13‐62)	85	776	7.49 ± 0.55 mm	7.47 ± 0.53 mm	Mean 99.6 (range 60–168)
Hansson et al. [25]	III	14 (13‐14)	24	169	8.1 ± 0.8*	8.1 ± 0.7*	Mean 82.8 ± 16.8 (range 60–108)
Daehlin et al. [18]	III	2ACL 24 ± 8 S 29 ± 10	68	628	8.4 ± 0.7*	8.2 ± 0.7 mm*	Median 156 (range 96–228)
Webster et al. [78]	III	28.5 ± 9.9	26	529	8.3 mm* <7 mm = 8 7 mm = 20 7.5 mm = 91 8 mm = 133 8.5 mm = 142 9 mm = 77 >9 mm = 58***	8.6 mm* <7 mm = 2 7 mm = 0 7.5 mm = 0 8 mm = 5 8.5 mm = 9 9 mm = 6 >9 mm =4***	Mean 57.6 ± 13.2
Morgan et al. [50]	IV	16 (13‐18)	38	156	7.02 ± 0.57*	7.1 ± 0.71*	Mean 199.2 (range 180‐247.2)
Adams et al. [1]	III	29.2 ± 7.7	8	41	≥8 mm = 29 <8 mm = 12**	≥8 mm = 3 <8 mm = 5**	Mean 73.7
Boots et al. [10]	III	≤8 mm = 23.6 ± 6.9 >8 mm = 26.1 ± 8.1	18	211	≤8 mm = 89 >8 mm = 122**	≤8 mm = 8 >8 mm = 10**	Mean 46 ± 11 mean 43 ± 11
Ates et al. [5]	IV	26 (10.5) 27 (10)	29	290	≤7 mm = 42, >7 mm = 248**	≤7 mm = 16, >7 mm = 13**	Median 62.5 (IQR 20.3) Median 60 (IQR 27)
Mirzay an et al. [48]	III	17.9 ± 3.5	393	5,568	5 mm = 1*** 6 mm = 16 6.5 mm = 53 7 mm = 590 7.5 mm = 616 8 mm = 2,075 8.5 mm = 679 9 mm = 1069 9.5 mm = 201 10 mm = 236 10.5 mm = 21 11 mm = 11	5 mm = 0*** 6 mm = 3 6.5 mm = 0 7 mm = 58 7.5 mm = 57 8 mm = 152 8.5 mm = 48 9 mm = 54 9.5 mm = 8 10 mm = 12 10.5 mm = 0 11 mm = 1	Median 20.4 (IQR 13.2–38.4)
Helito et al. [26]	III	2ACL 30.2 ± 9.4 S 32.1 ± 8.7	32	425	8.1 ± 0.8	8.3 ± 0.9	36.4 ± 12.6, median 34 range 24–84
Mo et al. [49]	I	Traditional 34.1 ± 10.1 All inside 34.2 ± 9.5	8	89	8.48 mm	8.14 mm	24
Runer et al. [60]	II	2ACL 23.3 ± 9.5 S	10	35	8.0 mm 6.5 mm = 0 7.0 mm = 5 7.5 mm = 7 8.0 mm = 15 8.5 mm = 5 9.0 mm = 3 9.5 mm = 1	7.8 mm 6.5 mm = 1 7.0 mm = 1 7.5 mm = 1 8.0 mm = 6 8.5 mm = 1 9.0 mm = 0 9.5 mm = 0	78.9 ± 13.6
Patellar tendon autograft							
Deahlin et al. [18]	III	2 ACL 24 ± 8 S 29 ± 10	3	27	9.85 ± 0.59 mm	9.7 ± 1.7 mm	Median 156 (range 96–228)
Gudas et al. [23]	III	26.0	2	86	10 mm*	10 mm*	24
Morgan et al. [50]	IV	16 (13‐18)	4	44	9.64 ± 0.65 mm	9.5 ± 0.58 mm	Mean 199.2 (range 180–247.2)
Shelbourne et al. [65]	IV	21.4‐26	19	695	10 mm*	10 mm*	n.r
Vindfeld et al. [76]	III	2ACL 24.2 S 28.4	17	21	8.7 ± 0.8	8.5 ± 0.9	11.0 (2.2–26.4)
Marigi et al. [46]	III	16 (15‐18)	5	59	<10 mm = 12 ≥ 10 mm = 47	<10 mm = 2 ≥ 10 mm = 3	Median 70.8 (range 46.8–123.6)
Snaebjornsson et al. [67]	III	At injury 24.9 ± 9.3 At reconstruction 26.8 ± 9.7	35	1294	<10 mm = 433 ≥10 mm = 826	<10 mm = 7 ≥10 mm = 28	24
Hughes et al. [28]	III	20.7 ± 4.2	10	89	Mean 9.8 (range 7–11)	Mean 10 (range 9–11)	52.3 ± 30.3
Quadriceps tendon autograft							
Runer et al. [60]	II	28.9 ± 11.6	8	37	8.2 mm 7 mm =3 7.5 mm = 0 8.0 mm = 24 9.0 mm = 10	8.3 mm 7 mm = 0 7.5 mm = 1 8 mm = 4 9.0 mm = 3	78.9 ± 13.6
Hughes et al. [28]	III	23.2 ± 8.2	5	148	Mean 9.8 mm (range 7–15 mm)	Mean 9.5 mm (range 7–12 mm)	49.9 ± 27.8

*Note*: Presented as mean ± standard deviation or as median with range. Values are presented as mean ± SD or median (range).

Abbreviations: ACLR, anterior cruciate ligament reconstruction; DB, double bundle; LOE, level of evidence; n.r, not reported; S, survivals; SB, single bundle; SD, standard deviation; 2ACL, graft failure.

### Hamstring tendon autograft

#### Continuous analysis

**Figure 2:**
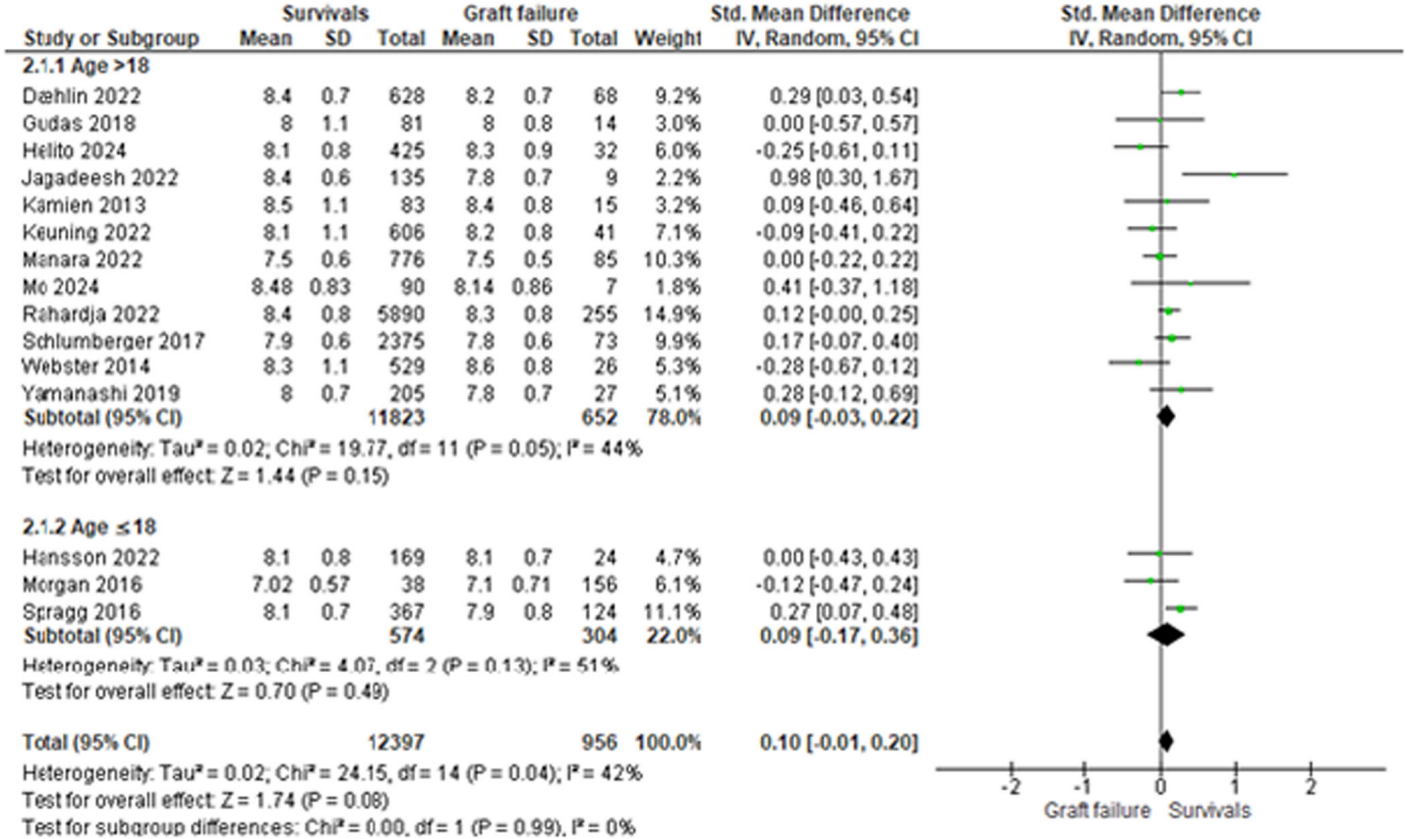
Standardised mean difference between patients who sustained a graft failure and patients who survived. CI, confidence interval; SD, standard deviation; Std., standard.

Four [23, 33, 35, 49] out of 15 [18, 23, 25, 26, 31, 33, 35, 44, 50, 57, 63, 69, 76, 78, 81] studies included in the meta‐analysis of mean autograft size had missing SD and therefore the SD was estimated based on the other included studies [22]. Mean autograft size for survivals was 8.19 ± 0.83 mm, based on 12,397 patients, and 7.91 ± 0.86 mm for graft failures based on 956 patients. There was no significant difference in autograft size between survivors and graft failures (SMD = 0.10 95% CI [−0.01 to 0.20], *p* = 0.08) (Figure 2). Certainty of evidence was deemed as low according to the GRADE assessment. Mean autograft size for patients > 18 years old was 8.20 ± 0.83 mm for survivals based on 11,823 patients, and 8.10 ± 0.79 mm for graft failures based on 652 patients. For patients ≤ 18 years old, mean autograft size for survivals was 8.03 ± 0.77 mm based on 574 patients, and 7.50 ± 0.86 mm for graft failures based on 304 patients. Analyses were non‐significant for the sub‐analysis on patient age.

#### Dichotomous analysis

There was a total of 22 studies included in the dichotomous analysis on HT autografts [1, 5, 10, 24, 29, 31, 32, 33, 43, 46, 47, 48, 52, 55, 57, 59, 67, 69, 73, 77, 78, 79].

**Figure 3:**
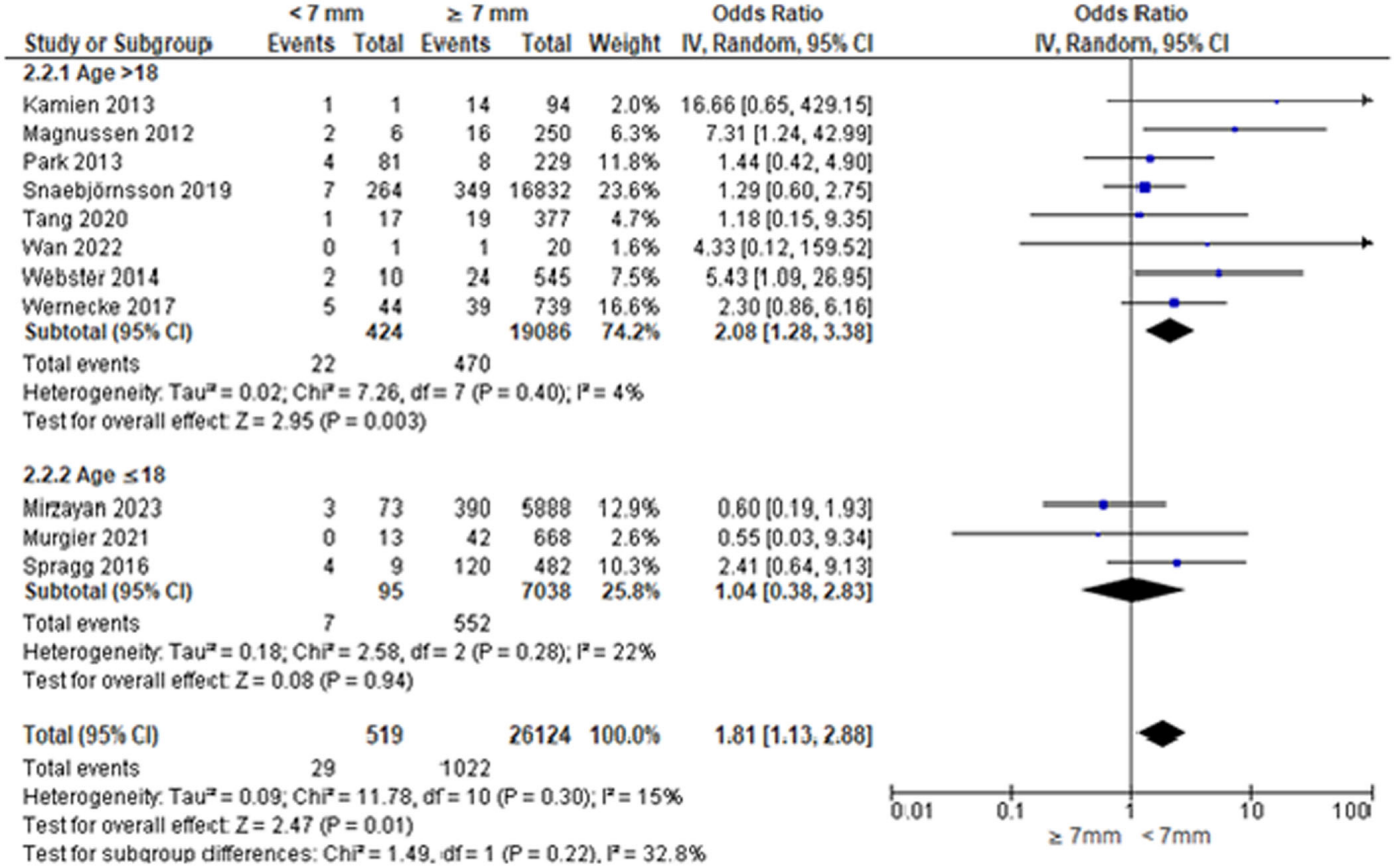
Odds ratio for a graft failure in patients with autograft size ≥ 7 and < 7 mm. CI, confidence interval.

Patients with autograft size < 7 mm had 81% significantly greater odds of graft failure compared to patients with ≥ 7 mm (OR = 1.81 95% CI [1.13–2.88], *p* = 0.01). In patients > 18 years of age have twice as likely to sustain a graft failure with < 7 mm graft diameter compared to ≥ 7 mm. Certainty of the evidence was deemed as very low (Figure 3). Patients with autograft size ≤ 7 mm had significantly greater odds for graft failure compared to > 7 mm (OR=1.80 95% CI [1.35–2.40] *p* < 0.0001). Certainty of evidence was deemed as very low for ≥ 7 mm and low for ≤ 7 mm. Age ≤ 18 years old had significantly greater odds for graft failure for autograft size ≤ 7 mm versus > 7 mm (*p* = 0.002) (Supporting Information: Appendix Figure 2).

**Figure 4:**
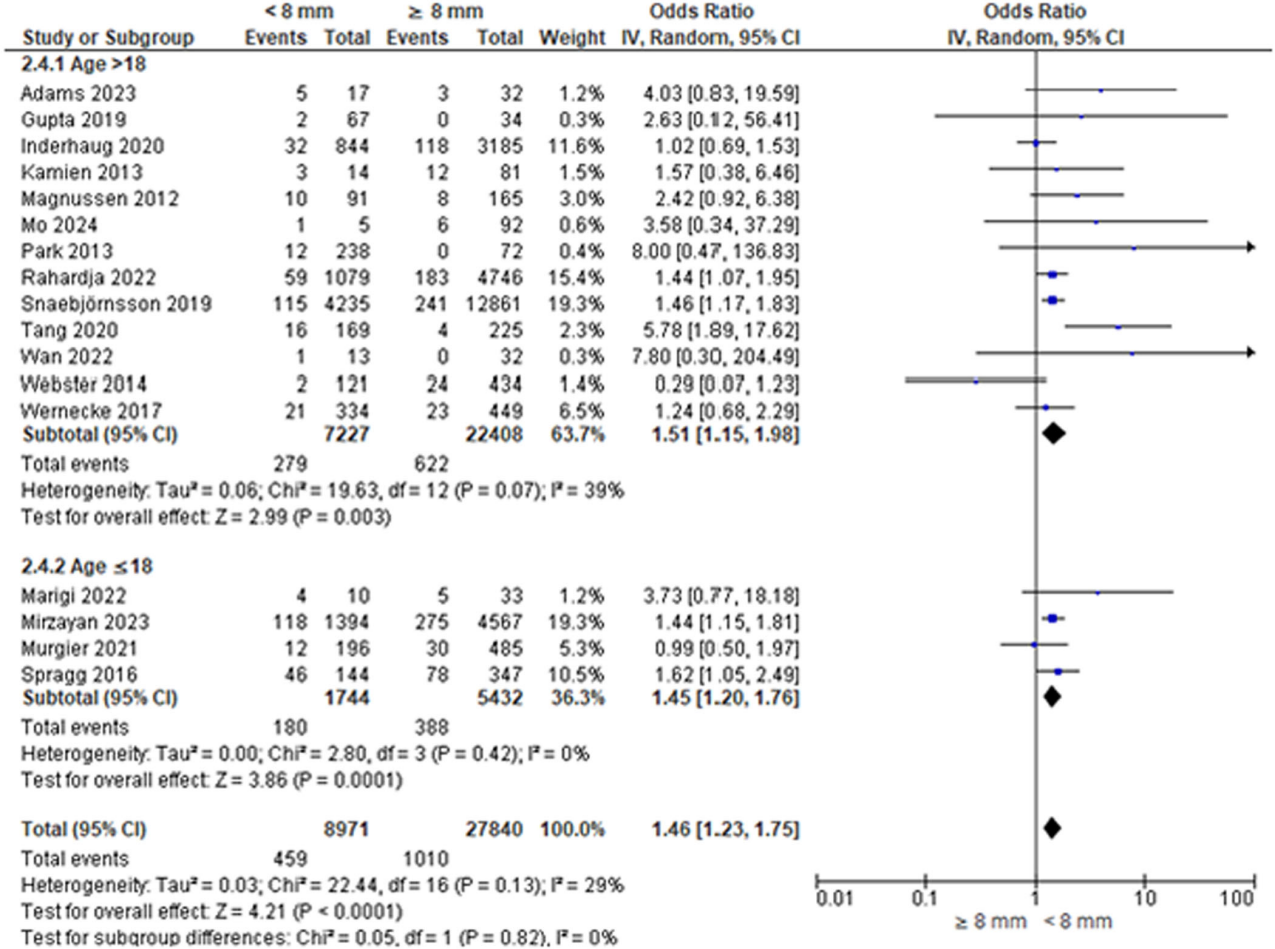
Odds ratio for a graft failure in patients with a autograft size ≥ 8 mm compared to <8 mm. CI, confidence interval.

Patients with an autograft size < 8 mm had 46% greater odds for graft failure compared to patients with ≥ 8 mm (OR = 1.46 95% CI [1.23–1.75], *p* = <0.0001) (Figure 4). Patients with autograft size ≤ 8 mm had significantly greater odds for graft failure (OR = 1.37 95% CI [1.15–1.63], *p* = 0.0004) compared to patients with autograft size > 8 mm (Supporting Information: Appendix Figure 3). Odds ratio remained significant in sub‐analyses based on age. Certainty of evidence was deemed very low for both analyses.

**Figure 5:**
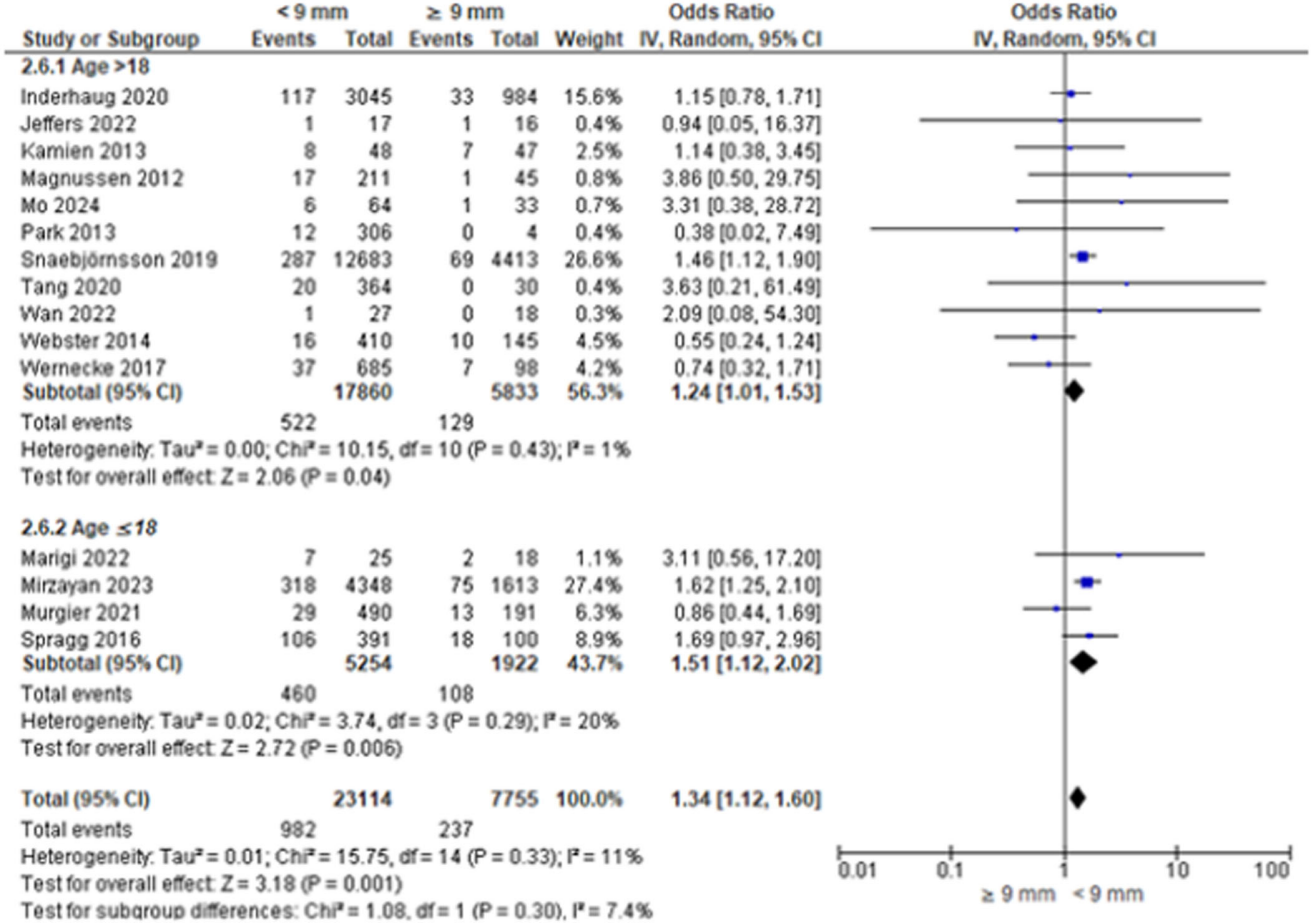
Odds ratio for a graft failure in patients with a autograft size ≥9 mm compared to <9 mm. CI, confidence interval.

Patients who had autograft size < 9 mm had 34% greater odds for graft failure compared to patients with ≥ 9 mm (OR = 1.34 95% CI [1.12–1.60], *p* = 0.001) (Figure 5). Patients with autograft size ≤ 9 mm had significantly greater odds for graft failure (OR = 1.35 95% CI [1.03–1.77], *p* = 0.03) compared to patients with autograft size > 9 mm (Supporting Information: Appendix Figure 4). Certainty of evidence for were deemed moderate for both analyses. Sub‐analysis on age remained significant.

**Figure 6:**
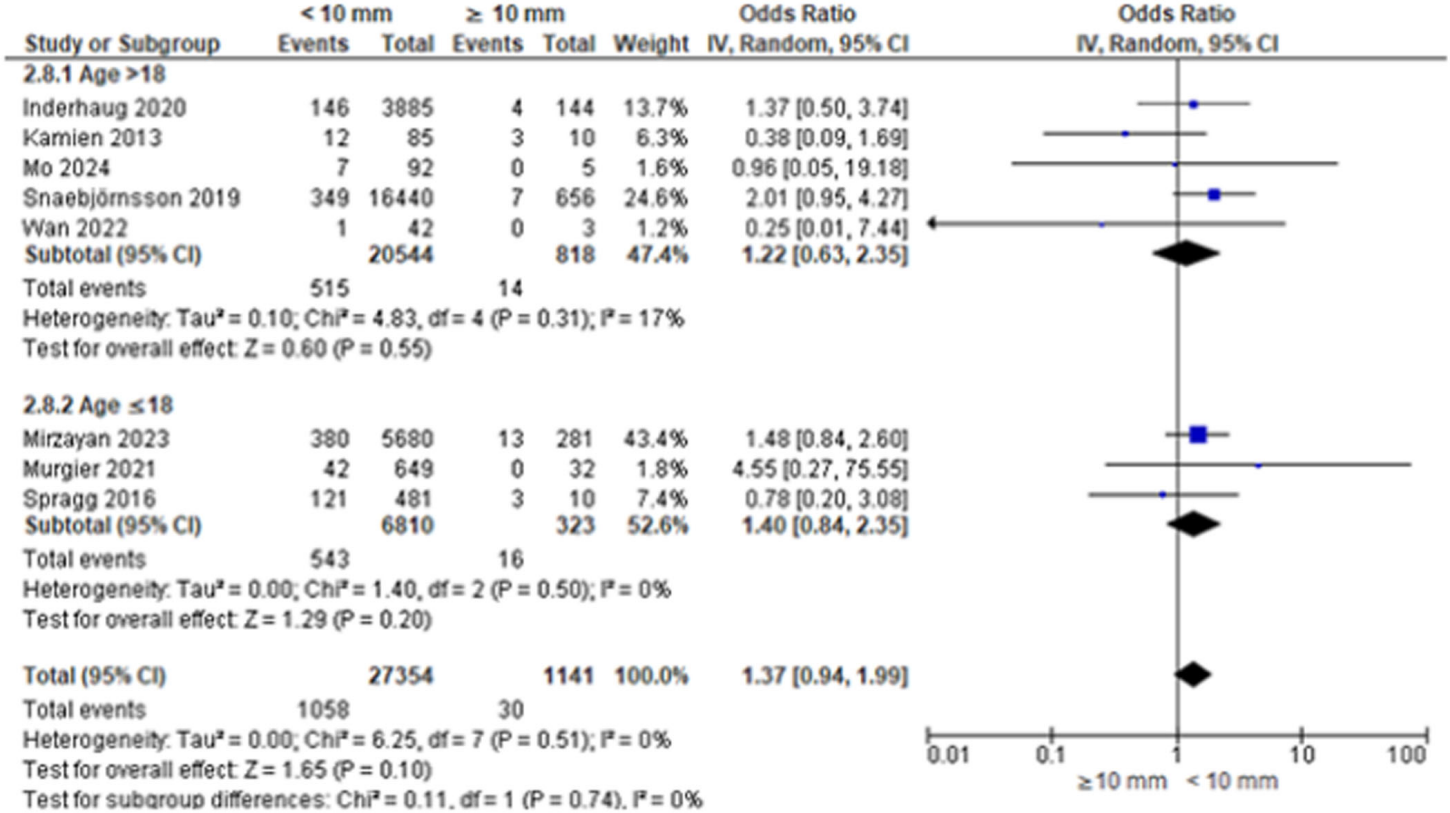
Odds ratio for a graft failure in patients with a autograft size ≥10 mm compared to <10 mm. CI, confidence interval.

No significant odds for graft failure were observed for patients with autograft size ≥ 10 mm compared to < 10 mm (Figure 6), or for autograft size > 10 mm compared to ≤ 10 mm (Supporting Information: Appendix Figure 5). Certainty of evidence was deemed moderate for ≥ 10 mm and very low for ≤ 10 mm. Analyses were non‐significant when sub‐analyses on ages were performed.

#### Risk of bias

The risk of bias assessment was made for each study included in this systematic review with RoBANS 2 for non‐randomised trials (Supporting Information: Appendix Table 1) and RoB 2 for randomised controlled trials (Supporting Information: Appendix Table 2). For most studies included in the HT autograft analysis, the risk of bias was deemed as low, with the highest risk of bias in the “incomplete outcome data” domain (19.3%). Sixteen (53%) of studies included in the HT autograft analysis were deemed to have low risk of bias.

#### Certainty of evidence

The GRADE was used to assess the certainty of evidence for each outcome included in this meta‐analysis. The GRADE was deemed “very low” to “moderate” for the analyses of the HT autografts presented in this systematic review. Certainty of evidence was downgraded to a baseline of 2 points for all studies but Mo et al. [49] due to a non‐RCT study design. The main reason for downgrading a study besides not being an RCT, was publication bias, where five out of nine analyses were downgraded −1, and imprecision which was downgraded for two out of nine analyses with −1 to −2 units.

### Patellar tendon autograft

#### Continuous analysis

**Figure 7:**
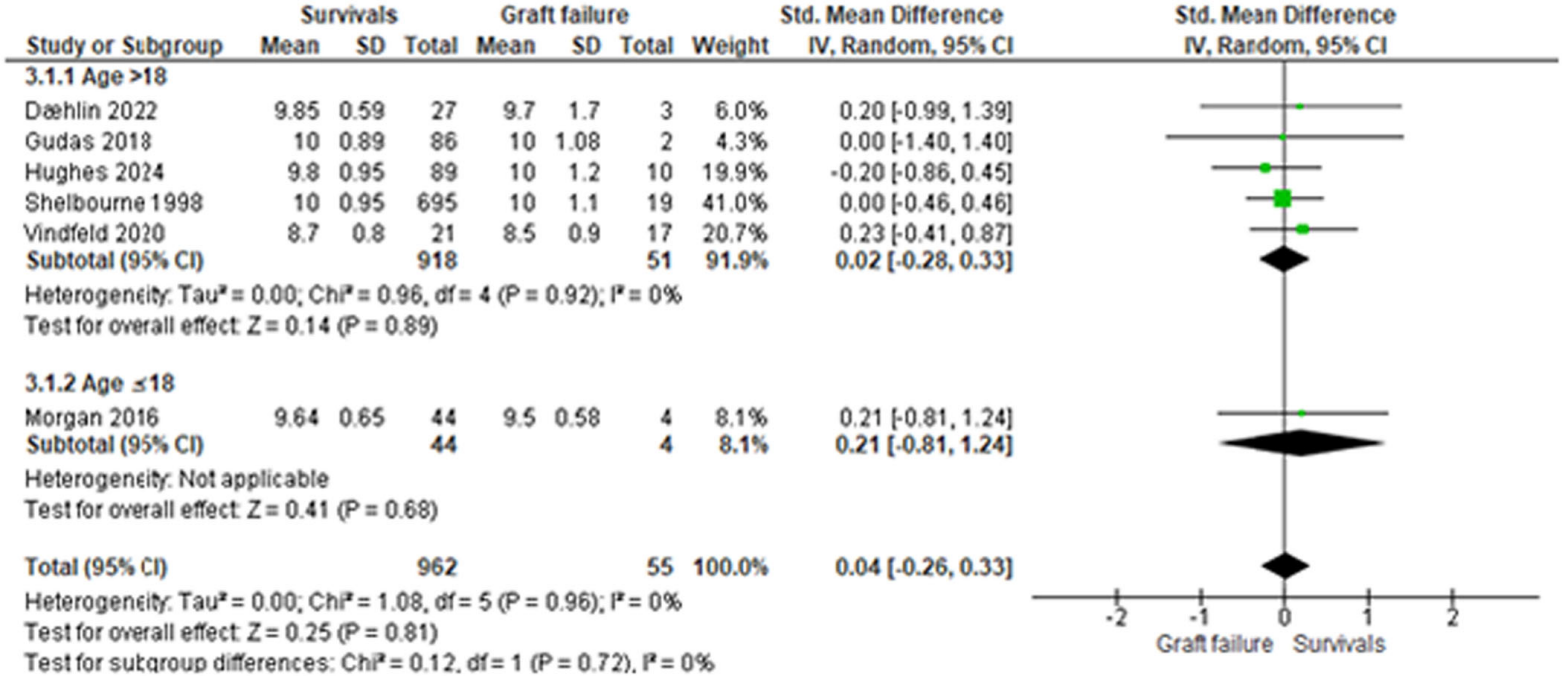
Pooled standardised mean difference for patients treated with patella tendon autograft for survivals and patients who sustained a graft failure. ACL, anterior cruciate ligament; CI, confidence interval; SD, standard deviation; Std., standardised.

Nine studies analysed the PT autograft [18, 23, 28, 46, 50, 52, 65, 67, 76]. Six studies could be pooled for continuous data (Figure 7) [18, 23, 28, 50, 65, 76], where three of these had missing SD [23, 28, 65], and three studies could be pooled for dichotomous data (Supporting Information: Appendix Figure 6) [18, 46, 67]. The mean autograft size for survivals was 9.93 ± 0.94 mm based on 962 patients, and 9.48 ± 1.22 mm for graft failures based on 55 patients. There was no significant difference in autograft size between patients who survived or sustained a graft failure (SMD = 0.04 95% CI [−0.26 to 0.33], *p* = 0.81). The certainty of evidence was very low. The sub‐analysis was non‐significant when analysing age ≥ 18 years versus < 18 years.

### Dichotomous analysis

Autograft size <10 versus ≥10 mm did not significantly influence the odds for graft failure (Supporting Information: Appendix Figure 6). The certainty of evidence was very low.

#### Risk of bias

The risk of bias assessment was made with the RoBANS 2 for non‐randomised trials (Supporting Information: Appendix Table 1). For studies included in the PT autograft analysis, the “incomplete outcome data” and “target group selection” were the only domains with high risk of bias. The overall risk of bias was deemed “low” in four of included studies (50%).

#### Certainty of evidence

The GRADE was used to assess the certainty of evidence for each outcome included in this meta‐analysis. The certainty of evidence for the PT autograft analysis in this systematic review was deemed as “very low”. Certainty of evidence was downgraded to a baseline of 2 points since none of the included studies were RCTs. The main reason for downgrading a study besides not being an RCT, was imprecision which was downgraded for two out of two analyses with −1 to −2 units. The other reason for down grading was publication bias, where two out of two analyses were downgraded −1.

### Quadriceps tendon autograft

There were only two studies that presented the QT as the chosen autograft [28, 60], without presenting standard deviation for any autograft size. Thus, these studies could not be included in a meta‐analysis. Patients received autograft sizes with a mean of 8.2 mm [60], and 9.8 mm [28] for survivals and, 8.3 mm [60] and 9.5 mm [28] for graft failures. In total, 13 graft failures were reported, which accounted for 6.6% of the total cohort. The follow‐up time ranged between 22.1 months and 92.5 months. All studies presented data for adult patients, mean age for Runer et al. [60] 28.9 ± 11.6 years for the full cohort and, Hughes et al. [28] 23.2 ± 8.2 years for the total cohort.

#### Risk of bias

The risk of bias assessment was made with the RoBANS 2 for non‐randomised trials (Supporting Information: Appendix Table 1). For studies included in the QT autograft analysis, the “comparability of target group”, “confounders”, “blinding of assessors” and “outcome assessment” were domains with high risk of bias. The overall risk of bias was deemed “low” in one of included studies (33%).

## DISCUSSION

Main findings of this systematic review and meta‐analysis were, with a general low certainty of evidence, that (1) there were no statistically significant difference in the mean autograft size between patients who survived and patients who sustained a graft failure independent of autograft, and (2) odds for a graft failure were significantly reduced for autograft size up to ≥ 9 mm compared to < 9 mm in patients reconstructed with HT autograft.

### Previous research

Prior to our systematic review and meta‐analysis, four other systematic reviews on the topic were published [3, 14, 30, 34]. Alomar et al. [3] reported that HT autograft with a autograft size ≥ 7 mm was significantly associated with lower odds for graft failure compared to <7 mm based on 15 studies with a total of 19,799 patients. Itoh et al. [30] reported a significantly reduced risk for graft failure for every 0.5 mm increase in HT autograft size from <7.0 to >9.0 mm based on five studies with a total of 19,333 patients. Conte et al. [14] reported that HT autografts size ≤8 mm had 6.8 times greater relative risk for graft failure compared to HT autografts size of >8 mm based on four studies with a total of 913 patients. Kang et al. [34] reported a significantly lower relative risk for graft failure with an HT autograft size ≥7 mm compared to an HT autograft size <7 mm. However, no significant difference was observed if the autograft cut‐off was set to 8 mm [34]. The present systematic review and meta‐analysis has a more comprehensive inclusion of patients treated with ACLR, including 35 studies with 46,268 patients, which covers both patients treated with HT, PT and QT autografts, and of all ages. Our results are in line with previous research, indicating that an HT autograft size up to ≥9 mm compared to <9 mm reduces the odds for a graft failure. Collectively, a large number of data supports the notion that ensuring a sufficient large autograft size, tailored to the unique anatomical space within the knee of each patient, is of importance for reducing the odds for graft failure in patients treated with ACLR using HT autograft.

### Autograft size

The mechanical strength capacity of a graft, that is, the tensile strength of the graft until failure, is related to the autograft size. Boniello et al. [9] reported that the mean load until failure was 2359 ± 474 N for 6 mm HT autografts, 3263 ± 677 N for 7 mm HT autografts, 3908 ± 556 N for 8 mm HT autografts, and 4360 ± 606 N for 9 mm HT autografts. A larger autograft size contains higher collagen content, which in turn enhances the autograft's mechanical ability to withstand tensile stress before failure [59]. The present study found that the odds for a graft failure were greater in smaller HT autograft sizes, ranging from 81% greater odds of a graft failure for <7 mm to 34% for <9 mm. Which is in line with previous studies that suggest likelihood of patients sustaining a graft failure has been reported to decrease for every 0.5 mm increase in autograft size [70]. The PT autograft has been reported with lower tensile strength for a harvested 10 mm bone block (between 2684 and 3397 N) [15], which could reflect the non‐significant results presented in our study for PT autograft. It is further noted that a nominal graft width reported for the PT autografts usually reflects bone block width rather than the tendon width considerably smaller, as a recent MRI‐based study in adolescents demonstrated that a “10 mm” PT autograft had a tendinous component measuring 6.3  ±  0.5 mm [7]. Importantly, the HT autograft is round, which allows a diameter measurement, while the PT autograft is flat and thus measured in width. Nonetheless, there are several aspects to account for about autograft size. Some studies only included patients ≤ 18 years [25, 45, 48, 50, 52], thus, younger, especially skeletally immature patients often have smaller notch sizes and therefore receive a smaller autograft size compared to older patients and thereby may have greater risk for graft failure [43]. Autograft inserted into skeletally immature patients does not grow [56], which implies that autografts might become too small as the patient grows. In the present study, 12% of included studies included patients <18 years old [25, 46, 50, 52]. The anatomical space of every unique patient should be considered, as too‐thick autograft size may contribute to knee impingement, especially in younger patients [74]. Thus, in an attempt to take skeletal maturity into account, we performed sub‐analyses for studies that included patients with a mean age of ≤ 18 years old. In sub‐analyses on age, statistical significance was observed up to 9 mm for patients ≤ 18 years, however, studies were few, with small sample sizes and few events, which resulted in large confidence intervals and very low certainty of evidence. Future studies should specifically investigate the impact of graft size for graft failure in the younger patients.

Another aspect of the tensile strength of the autograft is the maturation process of the graft. Remodelling of the tendon to become a functioning ligament can take 24 months until full resilience [53]. Thereby, an ACLR may infer a weaker autograft up to 24 months after reconstruction, which can predispose patients to greater risk for graft failure, especially with smaller autograft size. Among the included studies, eight studies had a mean of <24 months follow‐up [24, 31, 43, 48, 69, 76, 77, 81], and may thus be confounded by follow‐up time.

### Graft choice

In this systematic review there were 26 studies that presented data for HT autografts, five studies presented data for both HT and PT autografts, one study for HT and QT, two study for PT autograft, one study for PT and QT autograft. The lack of QT autograft studies reflects the current choice of autograft for primary ACLR, with a peak of 10% QT in 2018 [4]. The QT autograft has shown similar failure rates as for the HT and PT autograft but has been reported with superior functional outcomes, for example, mean Lysholm score compared to the HT autograft and less harvest site pain compared to PT autografts [51]. Results from the present systematic review and meta‐analysis noticed reduced odds for graft failure with greater autograft size up to ≥9 mm for HT autografts, however, non‐significant for 10 mm PT autografts. On the other hand, the difference in mean autograft size between survivals and graft failure for HT was 0.28 ± 0.03 mm and 0.45 ± 0.28 mm for PT. Although patients who survived had greater mean HT and PT autograft size compared to graft failures among the included studies, the differences were non‐significant, and the effect sizes were trivial both for HT autograft (SMD = 0.10) and for PT autograft (SMD = 0.04). Surgeons may need to consider adding a lateral extra‐articular tenodesis for autografts < 9 mm HT to reduce the risk for graft failure [11]. Although, the majority of previous research on graft failures are performed on HT autograft and further research is needed for the PT and QT autografts in order to draw conclusions from these autografts.

### Limitations

This systematic review and meta‐analysis was not without limitations. First, certainty of evidence for included studies was deemed as “very low” to “moderate” which indicate an uncertainty in the estimated effect. This is partly due to the lack of RCTs in the literature. However, based on the present meta‐analysis and previous results in the literature, it may be considered unethical to perform a RCT which compares graft sizes for graft failure. Second, we grouped graft failure and revision for some endpoints, which may be the reason for the large deviation in follow‐up time in included studies. Third, young patients may have smaller notches and thus receive a smaller autograft size. To adjust for this, we performed sub‐analyses based on age ≤ 18. On the same topic, larger patients with greater height or high body mass index might receive a larger autograft which was not accounted for in our analysis.

Fourth, there is no standardised way of measuring autograft size. Some orthopaedic surgeons like “tight” autograft whilst others want space in the tunnel for the screw. It is not certain that all surgeons use the measuring block but report the size of the tunnel, and there are different perceptions of how this is reported into registries. In addition, the included studies on PT autograft may have reported the size of the bone plug which is often larger than the tendon portion. This may limit the results and conclusions for the PT autograft. Furthermore, generalisability may be limited to the younger population who undergo ACLR with HT autograft.

Fifth, and in addition to autograft size, surgeons need to take other factors into account when performing reconstructive surgery, such as known risk factors as age, sex, type of activity, pre‐injury activity level, bony morphology, and concomitant injures such as ligament or meniscal injures amongst others. These known confounding risk factors were not considered in this review. In previous literature, the paediatric patients face substantial ethical, logistical and engagement barriers to trial enrolment [8], and despite a greater risk of re‐injury compared to male patients, females are underrepresented in orthopeadic research [17]. The choice of autograft size might merely be one of many factors influencing the probability of graft failure. Furthermore, differences in surgical techniques may influence graft failure rates, thus, the choice of graft type may be surgeon dependent. Outcomes related to return to pre‐injury activity such as muscle strength, hop performance, or rehabilitation programs were not considered for this systematic review and meta‐analysis [38, 39, 40, 66]. Lastly, our reliance on ClinicalTrials.gov may limit the comprehensiveness of our search, as not all trials are registered in this database and registered trials may be subject to selective reporting, incomplete data, or geographic bias.

## CONCLUSION

Patients treated with a greater HT autograft size have lower odds for a graft failure compared to patients with a smaller HT autograft size. There was no association between autograft size for patients treated with PT autograft and graft failure. Surgeons should consider the autograft size at the time of ACLR as the size of HT autograft influences the risk for a graft failure.

## AUTHOR CONTRIBUTIONS

Rebecca Hamrin Senorski, Kevin Teow, Kristian Samuelsson and Eric Hamrin Senorski were responsible for the design and outline of the study. Rebecca Hamrin Senorski was in charge of writing the first draft of the manuscript. Johan Högberg, Anna Nordenholm and Kevin Teow contributed majorly to data extraction and author Johan Högberg assisted greatly in the analysis. Rebecca Hamrin Senorski and Johan Högberg finalised the manuscript and all authors contributed to the preparation of the manuscript and revision of finalisation for submission. Janina Kaarre, Thorkell Snaebjörnsson, Volker Musahl, Kristian Samuelsson and Eric Hamrin Senorski made large contributions to the finalising of the manuscript for submission. All authors were responsible for accepting the final draft. Eric Hamrin Senorski is responsible for the design concept.

## CONFLICT OF INTEREST STATEMENT

Volker Musahl reports a relationship with Smith and Nephew Inc that includes: consulting or advisory, funding grants, and speaking and lecture fees and, have a relationship with Arthrex Inc that includes: funding grants. Dr. Musahl also reports a relationship with International Society of Arthroscopy, Knee Surgery and Orthopaedic Sports Medicine (ISAKOS) that includes: board membership. Dr. Musahl has patent #9,949,684 issued to U.S. Patent and, is deputy editor‐in‐chief of Knee Surgery, Sports Traumatology, Arthroscopy (KSSTA). Kristian Samuelsson reports a relationship with Getinge AB that includes: board membership. Eric Hamrin Senorski is the associate editor of *Journal of Orthopeadic and Sports Physical Therapy*.

## ETHICS STATEMENT

The authors have nothing to report.

## Supporting information

Appendix Table 1.

Appendix table 2.

KSSTA Appendix Figure 1.

KSSTA Appendix Figure 2.

KSSTA Appendix Figure 3.

KSSTA Appendix Figure 4.

KSSTA Appendix Figure 5.

KSSTA Appendix Figure 6.

Supporting file 1.

Supporting file 2.

## Data Availability

The authors have nothing to report.

## References

[1] Adams BG , Nowak MJ , Egan AC , Donohue MA , Galvin JW , Arrington ED . Autograft‐only and allograft‐augmented hamstring autograft have similar failure rates after anterior cruciate ligament reconstruction. Arthrosc Sports Med Rehabil. 2023;5(3):e725–e730. 37388891 10.1016/j.asmr.2023.03.015 PMC10300597

[2] Alkhalaf FNA , Hanna S , Alkhaldi MSH , Alenezi F , Khaja A . Autograft diameter in ACL reconstruction: size does matter. SICOT‐J. 2021;7:16–17. 33749586 10.1051/sicotj/2021018 PMC7984146

[3] Alomar AZ , Nasser ASB , Kumar A , Kumar M , Das S , Mittal S . Hamstring graft diameter above 7 mm has a lower risk of failure following anterior cruciate ligament reconstruction. Knee Surg Sports Traumatol Arthrosc. 2022;30(1):288–297. 33619635 10.1007/s00167-021-06503-0

[4] Arnold MP , Calcei JG , Vogel N , Magnussen RA , Clatworthy M , Spalding T , et al. ACL Study Group survey reveals the evolution of anterior cruciate ligament reconstruction graft choice over the past three decades. Knee Surg Sports Traumatol Arthrosc. 2021;29(11):3871–3876. 33486558 10.1007/s00167-021-06443-9

[5] Ateş O , Bozkurt I , Uluyardimci E , Öçgüder DA , Uğurlu M . Relationship between graft failure following anterior cruciate ligament reconstruction and hamstring autograft diameter. Acta Orthop Belg. 2023;89(3):429–433. 37935225 10.52628/89.3.11518

[6] Balshem H , Helfand M , Schünemann HJ , Oxman AD , Kunz R , Brozek J , et al. GRADE guidelines: 3. Rating the quality of evidence. J Clin Epidemiol. 2011;64(4):401–406. 21208779 10.1016/j.jclinepi.2010.07.015

[7] Beber SA , Jones RH , Cirrincione P , Gross PW , Green DW , Greditzer HG , et al. The intra‐articular tendinous graft diameter of 10‐mm bone‐patellar tendon‐bone autografts in adolescent patients: an MRI analysis of 100 patients. Orthop J Sports Med. 2024;12(8):23259671241264503. 39165331 10.1177/23259671241264503 PMC11334251

[8] Bencheva V , Mann NK , Rombey T , Pieper D , Schmiedl S . Barriers and facilitators to enrollment in pediatric clinical trials: an overview of systematic reviews. Syst Rev. 2024;13(1):283. 39568091 10.1186/s13643-024-02698-8 PMC11577732

[9] Boniello MR , Schwingler PM , Bonner JM , Robinson SP , Cotter A , Bonner KF . Impact of hamstring graft diameter on tendon strength: a biomechanical study. Arthroscopy. 2015;31(6):1084–1090. 25703286 10.1016/j.arthro.2014.12.023

[10] Boots N , van Melick N , Bogie R . A hamstring autograft diameter ≤ 8 mm is a safe option for smaller, lighter and female athletes who want to return to pivoting sports after ACL reconstruction: Results from a retrospective evaluation of a local ACL register. Knee Surg Sports Traumatol Arthrosc. 2023;31(12):5830–5836. 37943330 10.1007/s00167-023-07640-4

[11] Borque KA , Jones M , Laughlin MS , Balendra G , Willinger L , Pinheiro VH , et al. Effect of lateral extra‐articular tenodesis on the rate of revision anterior cruciate ligament reconstruction in elite athletes. Am J Sports Med. 2022;50(13):3487–3492. 36255290 10.1177/03635465221128828

[12] Ciccotti MC , Secrist E , Tjoumakaris F , Ciccotti MG , Freedman KB . Anatomic anterior cruciate ligament reconstruction via independent tunnel drilling: a systematic review of randomized controlled trials comparing patellar tendon and hamstring autografts. Arthroscopy. 2017;33(5):1062–1071.e5. 28359669 10.1016/j.arthro.2017.01.033

[13] Cohen J . Statistical Power Analysis for the Behavioral Sciences. 2nd ed. New York: Routledge; 1988.

[14] Conte EJ , Hyatt AE , Gatt CJ , Dhawan A . Hamstring autograft size can be predicted and is a potential risk factor for anterior cruciate ligament reconstruction failure. Arthroscopy. 2014;30(7):882–890. 24951356 10.1016/j.arthro.2014.03.028

[15] Cooper DE . Biomechanical properties of the central third patellar tendon graft: effect of rotation. Knee Surg Sports Traumatol Arthrosc. 1998;6(Suppl 1):S16–S19. 9608458 10.1007/s001670050217

[16] Cristiani R , Forssblad M , Edman G , Eriksson K , Stålman A . Age, time from injury to surgery and quadriceps strength affect the risk of revision surgery after primary ACL reconstruction. Knee Surg Sports Traumatol Arthrosc. 2021;29(12):4154–4162. 33661322 10.1007/s00167-021-06517-8 PMC8595184

[17] Crowell G , Adams J , Harmon I , Morey T , Long R , Vopat L , et al. Female athletes are underrepresented in anterior cruciate ligament reconstruction rehabilitation studies: a systematic review. Arthroscopy. 2025;41(8):3214–3222. 39536995 10.1016/j.arthro.2024.10.051

[18] Dæhlin L , Inderhaug E , Strand T , Parkar AP , Solheim E . The effect of posterior tibial slope on the risk of revision surgery after anterior cruciate ligament reconstruction. Am J Sports Med. 2022;50(1):103–110. 34792414 10.1177/03635465211054100 PMC8739589

[19] Dai W , Leng X , Wang J , Cheng J , Hu X , Ao Y . Quadriceps tendon autograft versus bone‐patellar tendon‐bone and hamstring tendon autografts for anterior cruciate ligament reconstruction: a systematic review and meta‐analysis. Am J Sports Med. 2022;50(12):3425–3439. 34494906 10.1177/03635465211030259

[20] Della Villa F , Buckthorpe M , Grassi A , Nabiuzzi A , Tosarelli F , Zaffagnini S , et al. Systematic video analysis of ACL injuries in professional male football (soccer): injury mechanisms, situational patterns and biomechanics study on 134 consecutive cases. Br J Sports Med. 2020;54(23):1423–1432. 32561515 10.1136/bjsports-2019-101247

[21] Frobell RB , Roos EM , Roos HP , Ranstam J , Lohmander LS . A randomized trial of treatment for acute anterior cruciate ligament tears. N Engl J Med. 2010;363(4):331–342. 20660401 10.1056/NEJMoa0907797

[22] Furukawa TA , Barbui C , Cipriani A , Brambilla P , Watanabe N . Imputing missing standard deviations in meta‐analyses can provide accurate results. J Clin Epidemiol. 2006;59(1):7–10. 16360555 10.1016/j.jclinepi.2005.06.006

[23] Gudas R , Jurkonis R , Smailys A . Comparison of return to pre‐injury sport after 10 mm size bone‐patellar tendon‐bone (BPTB) versus 8 mm hamstring anterior cruciate ligament reconstruction: a retrospective study with a two‐year follow‐up. Med Sci Monit. 2018;24:987–996. 29453931 10.12659/MSM.904709 PMC6354639

[24] Gupta R , Kapoor A , Soni A , Khatri S , Masih GD , Mittal N . Does hamstring tendon graft diameter affect the outcome of anterior cruciate ligament surgery? Sports Orthop Traumatol. 2019;35(2):166–170.

[25] Hansson F , Moström EB , Forssblad M , Stålman A , Janarv PM . Long‐term evaluation of pediatric ACL reconstruction: high risk of further surgery but a restrictive postoperative management was related to a lower revision rate. Arch Orthop Trauma Surg. 2022;142(8):1951–1961. 34459955 10.1007/s00402-021-04135-0 PMC9296415

[26] Helito CP , da Silva AGM , Sobrado MF , Guimarães TM , Gobbi RG , Pécora JR . Patients with more than 6.5° of knee hyperextension are 14.6 times more likely to have anterior cruciate ligament hamstring graft rupture and worse knee stability and functional outcomes. Arthroscopy. 2024;40(3):898–907. 37579954 10.1016/j.arthro.2023.07.057

[27] Higgins JPT . Measuring inconsistency in meta‐analyses. BMJ. 2003;327(7414):557–560. 12958120 10.1136/bmj.327.7414.557 PMC192859

[28] Hughes JD , Boden SA , Belayneh R , Dvorsky J , Mirvish A , Godshaw B , et al. Association of smaller intercondylar notch size with graft failure after anterior cruciate ligament reconstruction. Orthop J Sports Med. 2024;12(8):23259671241263883. 39165328 10.1177/23259671241263883 PMC11334253

[29] Inderhaug E , Drogset JO , Lygre SHL , Gifstad T . No effect of graft size or body mass index on risk of revision after ACL reconstruction using hamstrings autograft. Knee Surg Sports Traumatol Arthrosc. 2020;28(3):707–713. 30734062 10.1007/s00167-019-05395-5

[30] Itoh M , Itou J , Okazaki K , Iwasaki K . Estimation failure risk by 0.5‐mm differences in autologous hamstring graft diameter in anterior cruciate ligament reconstruction: a meta‐analysis. Am J Sports Med. 2024;52(2):535–543. 36876736 10.1177/03635465221150654

[31] Jagadeesh N , Dhawan T , Sheik F , Shivalingappa V . Does hamstring graft size affect functional outcome and incidence of revision surgery after primary anterior cruciate ligament (ACL) reconstruction? Cureus. 2022;14(1):e21158. 35165608 10.7759/cureus.21158 PMC8833284

[32] Jeffers KW , Shah SA , Calvert DD , Lemoine NP , Marucci J , Mullenix S , et al. High return to play and low reinjury rates in National Collegiate Athletic Association Division i football players following anterior cruciate ligament reconstruction using quadrupled hamstring autograft. Arthroscopy. 2022;38(1):99–106. 33957214 10.1016/j.arthro.2021.04.057

[33] Kamien PM , Hydrick JM , Replogle WH , Go LT , Barrett GR . Age, graft size, and Tegner activity level as predictors of failure in anterior cruciate ligament reconstruction with hamstring autograft. Am J Sports Med. 2013;41(8):1808–1812. 23813800 10.1177/0363546513493896

[34] Kang H , Dong C , Wang F . Small hamstring autograft is defined by a cut‐off diameter of 7 mm and not recommended with allograft augmentation in single‐bundle ACL reconstruction. Knee Surg Sports Traumatol Arthrosc. 2019;27(11):3650–3659. 30919001 10.1007/s00167-019-05475-6

[35] Keuning MC , Robben BJ , Brouwer RW , Stevens M , Bulstra SK , Zuurmond RG . Young men are at higher risk of failure after ACL hamstring reconstructions: a retrospective multivariate analysis. BMC Musculoskelet Disord. 2022;23(1):598. 35729572 10.1186/s12891-022-05547-8 PMC9210756

[36] Kim SJ , Choi CH , Kim SH , Lee SK , Lee W , Kim T , et al. Bone‐patellar tendon‐bone autograft could be recommended as a superior graft to hamstring autograft for ACL reconstruction in patients with generalized joint laxity: 2‐ and 5‐year follow‐up study. Knee Surg Sports Traumatol Arthrosc. 2018;26(9):2568–2579. 29502168 10.1007/s00167-018-4881-y

[37] Kim SY , Park JE , Lee YJ , Seo HJ , Sheen SS , Hahn S , et al. Testing a tool for assessing the risk of bias for nonrandomized studies showed moderate reliability and promising validity. J Clin Epidemiol. 2013;66(4):408–414. 23337781 10.1016/j.jclinepi.2012.09.016

[38] King E , Richter C , Daniels KAJ , Franklyn‐Miller A , Falvey E , Myer GD , et al. Biomechanical but not strength or performance measures differentiate male athletes who experience ACL reinjury on return to level 1 sports. Am J Sports Med. 2021;49(4):918–927. 33617291 10.1177/0363546520988018 PMC9677345

[39] King E , Richter C , Daniels KAJ , Franklyn‐Miller A , Falvey E , Myer GD , et al. Can biomechanical testing after anterior cruciate ligament reconstruction identify athletes at risk for subsequent ACL injury to the contralateral uninjured limb? Am J Sports Med. 2021;49(3):609–619. 33560866 10.1177/0363546520985283 PMC9938948

[40] Kyritsis P , Bahr R , Landreau P , Miladi R , Witvrouw E . Likelihood of ACL graft rupture: not meeting six clinical discharge criteria before return to sport is associated with a four times greater risk of rupture. Br J Sports Med. 2016;50(15):946–951. 27215935 10.1136/bjsports-2015-095908

[41] Landis JR , Koch GG . The measurement of observer agreement for categorical data. Biometrics. 1977;33(1):159–174. 843571

[42] Lawrence KW , Okewunmi JO , Chakrani Z , Cordero JK , Li X , Parisien RL . Randomized controlled trials comparing bone‐patellar tendon‐bone versus hamstring tendon autografts in anterior cruciate ligament reconstruction surgery are statistically fragile: a systematic review. Arthroscopy. 2024;40(3):998–1005. 37543146 10.1016/j.arthro.2023.07.039

[43] Magnussen RA , Lawrence JTR , West RL , Toth AP , Taylor DC , Garrett WE . Graft size and patient age are predictors of early revision after anterior cruciate ligament reconstruction with hamstring autograft. Arthroscopy. 2012;28(4):526–531. 22305299 10.1016/j.arthro.2011.11.024

[44] Manara JR , Salmon LJ , Kilani FM , Zelaya de Camino G , Monk C , Sundaraj K , et al. Repeat anterior cruciate ligament injury and return to sport in Australian Soccer Players after anterior cruciate ligament reconstruction with hamstring tendon autograft. Am J Sports Med. 2022;50(13):3533–3543. 36190172 10.1177/03635465221125467

[45] Marigi EM , Hale RF , Bernard CD , Bates N , Stuart MJ , Hewett TE , et al. Are 6‐month functional and isokinetic testing measures risk factors for second anterior cruciate ligament injuries at long‐T follow‐up? J Knee Surg. 2023;36(10):1060–1068. 35688443 10.1055/s-0042-1748824

[46] Marigi EM , Song BM , Wasserburger JN , Camp CL , Levy BA , Stuart MJ , et al. Anterior cruciate ligament reconstruction in 107 competitive wrestlers: outcomes, reoperations, and return to play at 6‐year follow‐up. Orthop J Sports Med. 2022;10(5):23259671221092770. 35547615 10.1177/23259671221092770 PMC9083060

[47] Mariscalco MW , Flanigan DC , Mitchell J , Pedroza AD , Jones MH , Andrish JT , et al. The influence of hamstring autograft size on patient‐reported outcomes and risk of revision after anterior cruciate ligament reconstruction: a Multicenter Orthopaedic Outcomes Network (MOON) Cohort Study. Arthroscopy. 2013;29(12):1948–1953. 24140144 10.1016/j.arthro.2013.08.025 PMC3844091

[48] Mirzayan R , Chang RN , Royse KE , Prentice HA , Maletis GB . No difference in revision risk between autologous hamstring graft less than 8 mm versus hybrid graft 8 mm or larger in anterior cruciate ligament reconstruction. Knee Surg Sports Traumatol Arthrosc. 2023;31(8):3465–3473. 37140654 10.1007/s00167-023-07437-5

[49] Mo IF , Harlem T , Faleide AGH , Strand T , Vindfeld S , Solheim E , et al. ACL reconstruction using quadrupled semitendinosus versus double‐stranded semitendinosus and gracilis autograft: 2‐year results from a prospective randomized controlled study. Am J Sports Med. 2024;52(8):1927–1936. 38845474 10.1177/03635465241254048

[50] Morgan MD , Salmon LJ , Waller A , Roe JP , Pinczewski LA . Fifteen‐year survival of endoscopic anterior cruciate ligament reconstruction in patients aged 18 years and younger. Am J Sports Med. 2016;44(2):384–392. 26759030 10.1177/0363546515623032

[51] Mouarbes D , Menetrey J , Marot V , Courtot L , Berard E , Cavaignac E . Anterior cruciate ligament reconstruction: a systematic review and meta‐analysis of outcomes for quadriceps tendon autograft versus bone‐patellar tendon‐bone and hamstring‐tendon autografts. Am J Sports Med. 2019;47(14):3531–3540. 30790526 10.1177/0363546518825340

[52] Murgier J , Powell A , Young S , Clatworthy M . Effectiveness of thicker hamstring or patella tendon grafts to reduce graft failure rate in anterior cruciate ligament reconstruction in young patients. Knee Surg Sports Traumatol Arthrosc. 2021;29(3):725–731. 32306133 10.1007/s00167-020-05973-y

[53] Nagelli CV , Hewett TE . Should return to sport be delayed until 2 years after anterior cruciate ligament reconstruction? Biological and functional considerations. Sports Med. 2017;47(2):221–232. 27402457 10.1007/s40279-016-0584-z PMC5226931

[54] Ouzzani M , Hammady H , Fedorowicz Z , Elmagarmid A . Rayyan‐a web and mobile app for systematic reviews. Syst Rev. 2016;5(1):210. 27919275 10.1186/s13643-016-0384-4 PMC5139140

[55] Park SY , Oh H , Park S , Lee JH , Lee SH , Yoon KH . Factors predicting hamstring tendon autograft diameters and resulting failure rates after anterior cruciate ligament reconstruction. Knee Surg Sports Traumatol Arthrosc. 2013;21(5):1111–1118. 22688502 10.1007/s00167-012-2085-4

[56] Pease F , Ehrenraich A , Skinner J , Williams A , Bollen S . The fate of the graft in ACL reconstruction in the skeletally immature. Orthop Proc. 2008;90–B(SUPP_III):576–577. 10.1302/0301-620X.90B4.19416 18378919

[57] Rahardja R , Love H , Clatworthy MG , Monk AP , Young SW . Suspensory versus interference tibial fixation of hamstring tendon autografts in anterior cruciate ligament reconstruction: results from the New Zealand ACL Registry. Am J Sports Med. 2022;50(4):904–911. 35048720 10.1177/03635465211070291

[58] Reinerink JM , Vendrig T , Keizer MNJ , Hoogeslag RAG , Brouwer RW . One type of graft for reconstruction of the ACL does not suit all patients based on their characteristics and sports: a scoping review. Musculoskelet Surg. 2025;109(2):115–125. 39249194 10.1007/s12306-024-00861-x PMC12122606

[59] Runer A , Keeling L , Wagala N , Nugraha H , Özbek EA , Hughes JD , et al. Current trends in graft choice for anterior cruciate ligament reconstruction – part I: anatomy, biomechanics, graft incorporation and fixation. J Exp Orthop. 2023;10(1):37. 37005974 10.1186/s40634-023-00600-4 PMC10067784

[60] Runer A , Suter A , Roberti di Sarsina T , Jucho L , Gföller P , Csapo R , et al. Quadriceps tendon autograft for primary anterior cruciate ligament reconstruction show comparable clinical, functional, and patient‐reported outcome measures, but lower donor‐site morbidity compared with hamstring tendon autograft: A matched‐pairs study with a mean follow‐up of 6.5 years. J ISAKOS Jt Disord Orthop Sports Med. 2023;8(2):60–67. 10.1016/j.jisako.2022.08.008 36216218

[61] Samuelsen BT , Webster KE , Johnson NR , Hewett TE , Krych AJ . Hamstring autograft versus patellar tendon autograft for ACL reconstruction: is there a difference in graft failure rate? A meta‐analysis of 47,613 patients. Clin Orthop Relat Res. 2017;475(10):2459–2468. 28205075 10.1007/s11999-017-5278-9 PMC5599382

[62] Sanders TL , Maradit Kremers H , Bryan AJ , Larson DR , Dahm DL , Levy BA , et al. Incidence of anterior cruciate ligament tears and reconstruction: a 21‐year population‐based study. Am J Sports Med. 2016;44(6):1502–1507. 26920430 10.1177/0363546516629944

[63] Schlumberger M , Schuster P , Schulz M , Immendörfer M , Mayer P , Bartholomä J , et al. Traumatic graft rupture after primary and revision anterior cruciate ligament reconstruction: retrospective analysis of incidence and risk factors in 2915 cases. Knee Surg Sports Traumatol Arthrosc. 2017;25(5):1535–1541. 26410092 10.1007/s00167-015-3699-0

[64] Seo HJ , Kim SY , Lee YJ , Park JE . RoBANS 2: a revised risk of bias assessment tool for nonrandomized studies of interventions. Korean J Fam Med. 2023;44(5):249–260. 37423253 10.4082/kjfm.23.0034 PMC10522469

[65] Shelbourne KD , Davis TJ , Klootwyk TE . The relationship between intercondylar notch width of the femur and the incidence of anterior cruciate ligament tears. Am J Sports Med. 1998;26(3):402–408. 9617403 10.1177/03635465980260031001

[66] Simonson R , Piussi R , Högberg J , Senorski C , Thomeé R , Samuelsson K , et al. Effect of quadriceps and hamstring strength relative to body weight on risk of a second ACL injury: a cohort study of 835 patients who returned to sport after ACL reconstruction. Orthop J Sports Med. 2023;11(4):23259671231157386. 37152619 10.1177/23259671231157386 PMC10155024

[67] Snaebjörnsson T , Hamrin‐Senorski E , Svantesson E , Karlsson L , Engebretsen L , Karlsson J , et al. Graft diameter and graft type as predictors of anterior cruciate ligament revision: a cohort study including 18,425 patients from the Swedish and Norwegian National Knee Ligament Registries. J Bone Jt Surg. 2019;101(20):1812–1820. 10.2106/JBJS.18.01467 31626005

[68] Snaebjörnsson T , Hamrin Senorski E , Ayeni OR , Alentorn‐Geli E , Krupic F , Norberg F , et al. Graft diameter as a predictor for revision anterior cruciate ligament reconstruction and KOOS and EQ‐5D values: a cohort study from the Swedish National Knee Ligament Register based on 2240 patients. Am J Sports Med. 2017;45(9):2092–2097. 28460194 10.1177/0363546517704177

[69] Spragg L , Chen J , Mirzayan R , Love R , Maletis G . The effect of autologous hamstring graft diameter on the likelihood for revision of anterior cruciate ligament reconstruction. Am J Sports Med. 2016;44(6):1475–1481. 27002103 10.1177/0363546516634011

[70] Spragg L , Chen J , Mirzayan R , Love R , Maletis G . The effect of autologous hamstring graft diameter on the likelihood for revision of anterior cruciate ligament reconstruction. Am J Sports Med. 2016;44(6):1475–1481. 27002103 10.1177/0363546516634011

[71] Sterne JAC , Savović J , Page MJ , Elbers RG , Blencowe NS , Boutron I , et al. RoB 2: a revised tool for assessing risk of bias in randomised trials. BMJ. 2019;366:l4898. 31462531 10.1136/bmj.l4898

[72] Stewart LA , Clarke M , Rovers M , Riley RD , Simmonds M , Stewart G , et al. Preferred reporting items for a systematic review and meta‐analysis of individual participant data: the PRISMA‐IPD statement. JAMA. 2015;313(16):1657–1665. 25919529 10.1001/jama.2015.3656

[73] Tang SP , Wan KH , Lee RH , Wong KK , Wong KK . Influence of hamstring autograft diameter on graft failure rate in Chinese population after anterior cruciate ligament reconstruction. Asia Pac J Sports Med Arthrosc Rehabil Technol. 2020;22:45–48. 32913712 10.1016/j.asmart.2020.07.005 PMC7453058

[74] Thein R , Spitzer E , Doyle J , Khamaisy S , Nawabi DH , Chawla H , et al. The ACL graft has different cross‐sectional dimensions compared with the native ACL: implications for graft impingement. Am J Sports Med. 2016;44(8):2097–2105. 27179055 10.1177/0363546516645531

[75] Tomita F , Yasuda K , Mikami S , Sakai T , Yamazaki S , Tohyama H . Comparisons of intraosseous graft healing between the doubled flexor tendon graft and the bone‐patellar tendon‐bone graft in anterior cruciate ligament reconstruction. Arthroscopy. 2001;17(5):461–476. 11337712 10.1053/jars.2001.24059

[76] Vindfeld S , Strand T , Solheim E , Inderhaug E . Failed meniscal repairs after anterior cruciate ligament reconstruction increases risk of revision surgery. Orthop J Sports Med. 2020;8(10):2325967120960538. 33195722 10.1177/2325967120960538 PMC7605002

[77] Wan KHM , Lai CYS , Tang SPK , Ng EPL , Moy RLT , Chan WL , et al. The use of five‐strand hamstring autograft to increase the graft size in anterior cruciate ligament reconstruction‐a prospective cohort study with satisfactory early clinical results. Arthrosc Sports Med Rehab. 2022;4(6):e1923–e1931. 10.1016/j.asmr.2022.07.010 PMC9791867 36579046

[78] Webster KE , Feller JA , Leigh WB , Richmond AK . Younger patients are at increased risk for graft rupture and contralateral injury after anterior cruciate ligament reconstruction. Am J Sports Med. 2014;42(3):641–647. 24451111 10.1177/0363546513517540

[79] Wernecke GC , Constantinidis A , Harris IA , Seeto BG , Chen DB , MacDessi SJ . The diameter of single bundle, hamstring autograft does not significantly influence revision rate or clinical outcomes after anterior cruciate ligament reconstruction. Knee. 2017;24(5):1033–1038. 28797872 10.1016/j.knee.2017.05.011

[80] Xie X , Liu X , Chen Z , Yu Y , Peng S , Li Q . A meta‐analysis of bone‐patellar tendon‐bone autograft versus four‐strand hamstring tendon autograft for anterior cruciate ligament reconstruction. Knee. 2015;22(2):100–110. 25547048 10.1016/j.knee.2014.11.014

[81] Yamanashi Y , Mutsuzaki H , Iwai K , Ikeda K , Kinugasa T . Failure risks in anatomic single‐bundle anterior cruciate ligament reconstruction via the outside‐in tunnel technique using a hamstring autograft. J Orthop. 2019;16(6):504–507. 31680741 10.1016/j.jor.2019.04.015 PMC6818388

