# Supplementary material for: Smaller hamstrings autograft size after primary ACL reconstruction is associated with higher odds for graft failure: A meta‐analysis on autografts sizes covering 46,268 patients
Source: Knee Surg Sports Traumatol Arthrosc. 2025 Sep 22;34(9):3212–32. doi: 10.1002/ksa.70069 (PMC13502458; doi:10.1002/ksa.70069)
Supplement: Supplementary file 2 [file KSA-34-3212-s002.docx]

| ***Appendix table 2. Assessment of risk of bias for included studies based on the Risk of Bias (RoB) 2.0.*** | | | | | | | | | | | | | | | | | | | | | | |
| --- | --- | --- | --- | --- | --- | --- | --- | --- | --- | --- | --- | --- | --- | --- | --- | --- | --- | --- | --- | --- | --- | --- |
|  | ***1.1*** | ***1.2*** | ***1.3*** | ***2.1*** | ***2.2*** | ***2.3*** | ***2.4*** | ***2.5*** | ***2.6*** | ***2.7*** | ***3.1*** | ***3.2*** | ***3.3*** | ***3.4*** | ***4.1*** | ***4.2*** | ***4.3*** | ***4.4*** | ***4.5*** | ***5.1*** | ***5.2*** | ***5.3*** |
| Mo et al.^49^ | Y | Y | N | PN | Y | N | - | - | PN | No | NI | PY | - | - | N | N | N | - | - | NI | PN | N |
| *Overall risk of bias* | Low | | | Some concerns | | | | | | | Low | | | | Low | | | | | Some concerns | | |

N; No, NI; No Information, PN; Probably No; PY; Probably Yes, Y; Yes
