# Supplementary material for: Smaller hamstrings autograft size after primary ACL reconstruction is associated with higher odds for graft failure: A meta‐analysis on autografts sizes covering 46,268 patients
Source: Knee Surg Sports Traumatol Arthrosc. 2025 Sep 22;34(9):3212–32. doi: 10.1002/ksa.70069 (PMC13502458; doi:10.1002/ksa.70069)
Supplement: Supplementary file 6 [file KSA-34-3212-s006.docx]

**SUPPLEMENTARY FILE 2**

**Title of the Manuscript**

Smaller hamstrings autograft size after ACL reconstruction is associated with higher odds for graft failure: a meta-analysis on autografts sizes covering 46,268 patients after primary ACL reconstruction

**Authors**

Rebecca Hamrin Senorski (PT, MSc)1,2, Kevin Teow (MD) 2,3, Johan Högberg (PT, MSc)1,2, Janina Kaarre (MD)2,3, Anna Nordenholm (PT, PhD)2, Thorkell Snaebjörnsson (MD)2,3,5, Volker Musahl (MD)4, Kristian Samuelsson (MD, PhD)2,3,5, Eric Hamrin Senorski (PT, PhD)1,2

**Affiliations**

1Unit of Physiotherapy, Department of Health and Rehabilitation, Institute of Neuroscience and Physiology, Sahlgrenska Academy, University of Gothenburg, Gothenburg, Sweden

2Sahlgrenska Sports Medicine Center, Gothenburg, Sweden

3Department of Orthopaedics, Institute of Clinical Sciences, Sahlgrenska Academy, University of Gothenburg, Gothenburg, Sweden.

4Department of Orthopaedic Surgery, UPMC Freddie Fu Sports Medicine Center, University of Pittsburgh Medical Center, Pittsburgh, PA, USA

5Department of Orthopaedics, Sahlgrenska University Hospital, Mölndal, Sweden

**Corresponding Author**

**Name: Rebecca Hamrin Senorski
Email:** [**rebecca.hamrin.senorski@gu.se**](mailto:rebecca.hamrin.senorski@gu.se)

Analysis 1 on HT autograft mean autograft size HT (d=0.11, 95% CI [-0.02;0.23] p=0.10). Removed Gudas 2018, Keuning 2022, Kamien 2013 and Mo 2024 from the analysis.

Analysis 2 on HT autograft, <7/≥7 mm (d=1.70, 95% CI [1.09;2.66] p=0.02). Removed Kamien 2013 from the analysis.

Analysis 3 on HT autograft, >7mm/≤7mm HT (d=1.80, 95% CI [1.33; 2.42] p=0.0001). Removed Kamien 2013 from the analysis.

Analysis 4 on HT autograft, <8mm/≥8mm HT (d=1.46, 95% CI [1.21; 1.76] p<0.0001). Removed Kamien 2013, and Mo 2024 from the analysis.

Analysis 5 on HT autograft, ≤8mm/>8mm HT (d=1.35, 95% CI [1.11; 1.63] p=0.002). Removed Kamien 2013 and Mo 2024 from the analysis.

Analysis 6 on HT autograft, <9mm/≥9mm HT (d=1.31, 95% CI [1.07; 1.60] p=0.008). Removed Kamien 2013 and Mo 2024 from the analysis.

Analysis 7 on HT autograft, >9/≤9mm HT (d=1.38, 95% CI [10.5; 1.83] p=0.02). Removed Kamien 2013 and Mo 2024 from the analysis.

Analysis 8 on HT autograft, <10/≥10mm HT (d=1.50, 95% CI [1.02; 2.22] p=0.04). Removed Kamien 2013 and Mo 2024 from the analysis.

Analysis 9 on HT autograft, >/≤10 mm HT (d=0.76, 95% CI [0.13; 4.30] p=0.75). Removed Kamien 2013 from the analysis.

Analysis 10 on PT mean autograft size (d=0.10, 95% CI [-0.23; 0.44] p=0.55). Removed Gudas 2018 and Hughes 2024 from the analysis.
