# Supplementary material for: Smaller hamstrings autograft size after primary ACL reconstruction is associated with higher odds for graft failure: A meta‐analysis on autografts sizes covering 46,268 patients
Source: Knee Surg Sports Traumatol Arthrosc. 2025 Sep 22;34(9):3212–32. doi: 10.1002/ksa.70069 (PMC13502458; doi:10.1002/ksa.70069)
Supplement: Supplementary file 8 [file KSA-34-3212-s008.docx]

**SUPPLEMENTARY FILE 1**

**Title of the Manuscript**

Smaller hamstrings autograft size after ACL reconstruction is associated with higher odds for graft failure: a meta-analysis on autografts sizes covering 46,268 patients after primary ACL reconstruction

**Authors**

Rebecca Hamrin Senorski (PT, MSc)1,2, Kevin Teow (MD) 2,3, Johan Högberg (PT, MSc)1,2, Janina Kaarre (MD)2,3, Anna Nordenholm (PT, PhD)2, Thorkell Snaebjörnsson (MD)2,3,5, Volker Musahl (MD)4, Kristian Samuelsson (MD, PhD)2,3,5, Eric Hamrin Senorski (PT, PhD)1,2

**Affiliations**

1Unit of Physiotherapy, Department of Health and Rehabilitation, Institute of Neuroscience and Physiology, Sahlgrenska Academy, University of Gothenburg, Gothenburg, Sweden

2Sahlgrenska Sports Medicine Center, Gothenburg, Sweden

3Department of Orthopaedics, Institute of Clinical Sciences, Sahlgrenska Academy, University of Gothenburg, Gothenburg, Sweden.

4Department of Orthopaedic Surgery, UPMC Freddie Fu Sports Medicine Center, University of Pittsburgh Medical Center, Pittsburgh, PA, USA

5Department of Orthopaedics, Sahlgrenska University Hospital, Mölndal, Sweden

**Corresponding Author**

**Name: Rebecca Hamrin Senorski
Email:** [**rebecca.hamrin.senorski@gu.se**](mailto:rebecca.hamrin.senorski@gu.se)

**ACL reconstruction**

((( "Anterior Cruciate Ligament"[Mesh] OR ACL OR (anterior AND cruciate AND ligament*))
AND
(surgical* OR surgery OR surgeries OR reconstruction* OR reconstructive OR reconstructed OR repair*))
OR
"Anterior Cruciate Ligament Reconstruction"[Mesh]
OR
ACLR)

**AND**

exp Reoperation/ OR exp Reinjuries/
OR
reoperat* OR re-operat* OR revision* OR rerupt* OR re-rupt* OR reinjur* or re-injur* OR repeat* OR second* OR subsequen*

**AND**

(("Autografts"[Mesh] OR "Transplantation, Autologous"[Mesh] OR "Transplants"[Mesh] OR "Transplantation"[Mesh]) AND (diameter OR size OR sizes OR sized OR width OR small* OR large* OR thin* OR thick*))
OR
 ((autograft* OR auto-graft* OR allograft* OR allo-graft* OR graft* OR autotransplant* OR auto-transplant* OR transplant*) adj4 (diameter OR size OR sizes OR sized OR width OR small* OR large* OR thin* OR thick*))

**NOT**

(comment or editorial or letter).pt.

**NOT**

(animals NOT (animals and humans)).sh.

**AND**

english, danish, finnish, norwegian, swedish
