# Supplementary material for: Smaller hamstrings autograft size after primary ACL reconstruction is associated with higher odds for graft failure: A meta‐analysis on autografts sizes covering 46,268 patients
Source: Knee Surg Sports Traumatol Arthrosc. 2025 Sep 22;34(9):3212–32. doi: 10.1002/ksa.70069 (PMC13502458; doi:10.1002/ksa.70069)
Supplement: Supplementary file 10 [file KSA-34-3212-s010.docx]

*Appendix Table 1:* *Assessment of risk of bias for included studies based on the Risk of Bias Assessment tool for Non-randomized Studies (RoBANS).*

|  | Comparability of the target group | Target group selection | Confounders | Measurement of intervention/  exposure | Blinding of assessors | Outcome assessment | Incomplete outcome data | Selective outcome reporting | Summarized bias |
| --- | --- | --- | --- | --- | --- | --- | --- | --- | --- |
| **Hamstrings tendon autograft** | |  |  |  |  |  |  |  |  |
| Gudas et al.[23] |  |  |  |  |  |  |  |  | L = 62.5%  U = 37.5% |
| Jagadeesh et al.[31] |  |  |  |  |  |  |  |  | L = 37.5%  U = 62.5% |
| Gupta et al.[24] |  |  |  |  |  |  |  |  | L = 50.0%  U = 37.5%  H = 12.5% |
| Murgier et al.[52] |  |  |  |  |  |  |  |  | L = 62.5%  U = 37.5% |
| Wernecke et al.[79] |  |  |  |  |  |  |  |  | L = 50.0%  U = 37.5%  H = 12.5% |
| Keuning et al.[35] |  |  |  |  |  |  |  |  | L = 62.5%  U = 37.5% |
| Rahardja et al.[57] |  |  |  |  |  |  |  |  | L = 62.5%  U = 37.5% |
| Mariscalco et al.[47] |  |  |  |  |  |  |  |  | L = 50.0%  U = 50.0% |
| Marigi et al.[46] |  |  |  |  |  |  |  |  | L = 50.0%  U = 50.0% |
| Snaebjornsson et al.[67] |  |  |  |  |  |  |  |  | L = 75.0%  U = 12.5%  H = 12.5% |
| Magnussen et al.[43] |  |  |  |  |  |  |  |  | L = 50.0%  U = 37.5%  H = 12.5% |
| Tang et al.[73] |  |  |  |  |  |  |  |  | L = 37.5%  U= 62.5% |
| Spragg et al.[69] |  |  |  |  |  |  |  |  | L = 62.5%  U = 25.0%  H = 12.5% |
| Kamien et al.[33] |  |  |  |  |  |  |  |  | L = 25.0%  U = 75.0% |
| Park et al.[55] |  |  |  |  |  |  |  |  | L = 25.0%  U= 62.5%  H = 12.5% |
| Inderhaug et al.[29] |  |  |  |  |  |  |  |  | L = 62.5%  U = 37.5% |
| Schlumberger et al.[63] |  |  |  |  |  |  |  |  | L = 62.5%  U = 37.5% |
| Jeffers et al.[32] |  |  |  |  |  |  |  |  | L = 12.5%  U = 37.5%  H = 50.0% |
| Yamanashi et al.[81] |  |  |  |  |  |  |  |  | L = 62.5%  U = 25.0%  H = 12.5% |
| Wan et al.[77] |  |  |  |  |  |  |  |  | L = 75.0%  U = 25.0% |
| Manara et al.[44] |  |  |  |  |  |  |  |  | L = 62.5%  U = 12.5%  H = 25.0% |
| Hansson et al.[25] |  |  |  |  |  |  |  |  | L = 75.0%  U = 25.0% |
| Daehlin et al.[18] |  |  |  |  |  |  |  |  | L = 75.0%  U = 25.0% |
| Webster et al.[78] |  |  |  |  |  |  |  |  | L = 37.5%  U = 37.5%  H = 25.0% |
| Morgan et al. [50] |  |  |  |  |  |  |  |  | L = 37.5%  U = 37.5%  H = 25.0% |
| Adams et al.[1] |  |  |  |  |  |  |  |  | L = 37.5%  U = 12.5%  H = 50.0% |
| Boots et al.[10] |  |  |  |  |  |  |  |  | L = 75.0%  U = 25.0% |
| Ates et al.[5] |  |  |  |  |  |  |  |  | L = 75.0%  U = 25.0% |
| Mirzayan et al.[48] |  |  |  |  |  |  |  |  | L =75.0%  U =12.5%  H = 12.5% |
| Helito et al.[26] |  |  |  |  |  |  |  |  | L=62.5%  U=25.0%  H=12.5% |
| Runer et al.[60] |  |  |  |  |  |  |  |  | L=25.0%  U= 25.0%  H=50.0% |
| Summarized bias | L= 64.5%  U = 29.0%  H = 6.5% | L= 87.1%  U = 3.3%  H = 9.6% | L= 12.9%  U = 74.2%  H = 12.9% | L= 80.6%  U = 9.7%  H = 9.7% | L = 6.5%  U = 87.0%  H = 6.5% | L = 80.0%  U = 12.9%  H = 16.1% | L = 35.5%  U = 45.2%  H = 19.3% | L = 74.2%  U = 19.3%  H = 6.5% |  |
| **Patellar tendon autograft** | | | |  |  |  |  |  |  |
| Daehlin et al.[18] |  |  |  |  |  |  |  |  | L = 75.0%  U = 25.0% |
| Gudas et al.[23] |  |  |  |  |  |  |  |  | L = 62.5%  U = 37.5% |
| Morgan et al.[50] |  |  |  |  |  |  |  |  | L = 37.5%  U = 37.5%  H = 25.0% |
| Shelbourne et al.[65] |  |  |  |  |  |  |  |  | L = 75.0%  U = 25.0% |
| Vindfeld et al.[76] |  |  |  |  |  |  |  |  | L = 62.5%  U = 37.5% |
| Marigi et al.[46] |  |  |  |  |  |  |  |  | L = 50.0%  U = 50.0% |
| Snaebjornsson et al.[67] |  |  |  |  |  |  |  |  | L = 75.0%  U = 12.5%  H = 12.5% |
| Hughes et al.[28] |  |  |  |  |  |  |  |  | L= 100.0%  U= 0.0%  H= 0.0% |
| Summarized bias | L=87.5%  U=12.5% | L=87.5%  H=12.5% | L=50.0%  U=50.0% | L=75.0%  U=25.0% | L=25.0%  U=75.0% | L=87.5%  U=12.5% | L=37.5%  U=37.5%  H=25.0% | L=87.5%  U=12.5% |  |
| **Quadriceps tendon autograft** | | | | | | | | | |
| Runer et al.[60] |  |  |  |  |  |  |  |  | L=25.0%  U= 25.0%  H=50.0% |
| Hughes et al.[28] |  |  |  |  |  |  |  |  | L= 100.0%  U= 0.0%  H= 0.0% |
|  | L=50.0%  H=50.0% | L=100.0% | L=50.0%  H=50.0% | L=100.0% | L=50.0%  H=50.0% | L=50.0%  H=50.0% | L=50.0%  U=50.0% | L=50.0%  U=50.0% |  |
| *Green; Low risk of bias, Yellow; Unclear risk of bias, Red; High risk of bias.* | | | | | | | | | |
