# Supplementary material for: Smaller hamstrings autograft size after primary ACL reconstruction is associated with higher odds for graft failure: A meta‐analysis on autografts sizes covering 46,268 patients
Source: Knee Surg Sports Traumatol Arthrosc. 2025 Sep 22;34(9):3212–32. doi: 10.1002/ksa.70069 (PMC13502458; doi:10.1002/ksa.70069)
Supplement: Supplementary file 11 [file KSA-34-3212-s001.docx]

**Appendix Fig. 4** Odds ratio for patients with autograft size ≤9 mm and >9 mm
